# Position Estimation Considering Uncertain Classification of Cyclists Based on Partially Observed Movement Characteristics

**DOI:** 10.3390/s26103146

**Published:** 2026-05-15

**Authors:** Kento Suzuki, Takuma Ito

**Affiliations:** Graduate School of Engineering, The University of Tokyo, 7-3-1 Hongo, Tokyo 113-8656, Japan

**Keywords:** movement estimation, movement characteristics, soft classification, extended Kalman filter, vulnerable road users, intelligent transportation system

## Abstract

Prevention of crossing collisions between cyclists and vehicles at nonsignalized intersections on community roads where walls and hedges limit visibility is required in Japan. Because available observation information in real-time is limited on community roads, the use of statistical information that represents the typical movement characteristics of cyclists is effective to compensate for the lack of observation information. From such a background, in our previous study, we proposed a method to construct “location-dependent statistical information” (LDSI) and a method to utilize it as “virtual observation” (VO) and “virtual control input” (VCI) in stochastic position estimation. Here, although LDSI was constructed for multiple clusters of cyclists, the classification method of the cyclists observed in real-time was not considered. In the real world, the limitation of the observation information causes classification uncertainty. Thus, in this study, we propose a position estimation method that utilizes soft classification results and considers classification uncertainty by integrating VO and VCI derived from LDSI of each cluster. To evaluate the proposed method in this study, we conduct a simulation and an experiment in the real world. Through the comparison with conventional methods, we confirm that our proposed method in this study improves the performance of the position estimation. The proposed method will contribute to a cooperative safety system.

## 1. Introduction

**Figure 1:**
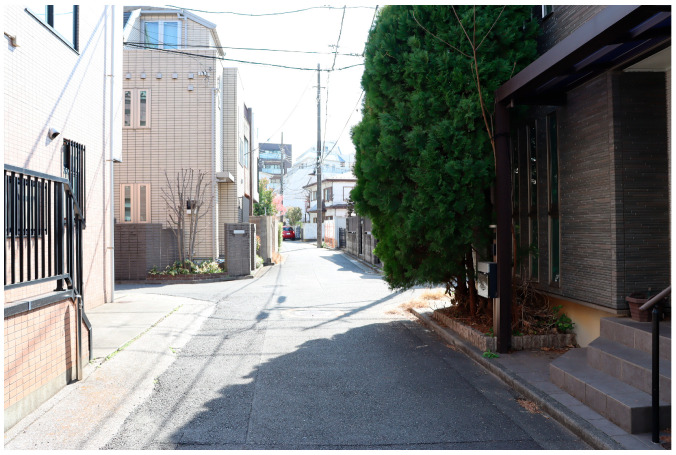
Typical intersection on a Japanese community road.

Although traffic accidents in Japan have been gradually decreasing in recent years [1], the decreasing rate of traffic fatalities on community roads is low [2]. Figure 1 shows the typical intersection on Japanese community roads where visibility is bad due to the walls and hedges, resulting in a high risk of traffic accidents around the intersections, such as crossing collisions. In fact, although the number of rear-end collisions of vehicles in Japan decreased by approximately 59% over the past 10 years, while that of crossing collisions decreased only approximately 43% [3]. Additionally, because of the relatively high velocity of cyclists, it is relatively hard to prevent cyclist-related accidents. Furthermore, their high speed sometimes results in severe damage. Thus, countermeasures are necessary for the prevention of cyclist-related accidents on community roads.

**Figure 2:**
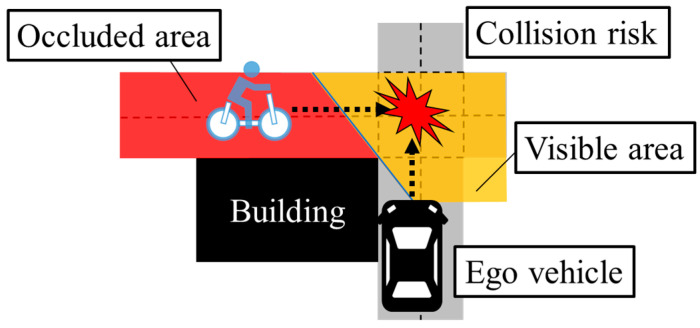
Conceptual schematic of visibility at intersections in community roads.

Advanced Driver Assistance System (ADASs) that depend on onboard sensors might not work adequately on community roads due to the low visibility at intersections, as shown in Figure 2. Thus, for preventing traffic accidents in such a traffic environment, grasping the position of cyclists in the occluded area in advance is important. Considering such a background, cooperative perception methods using roadside sensors [4,5,6] and methods using real-time information which portable devices of cyclists provide [7,8,9] can be considered as effective approaches for collision prevention on community roads. However, dense placement of roadside sensors is impractical due to the cost constraints. In addition, not all cyclists provide information from their portable devices. Therefore, the lack of real-time information is the challenging point in position estimation in the assumed situation.

**Figure 3:**
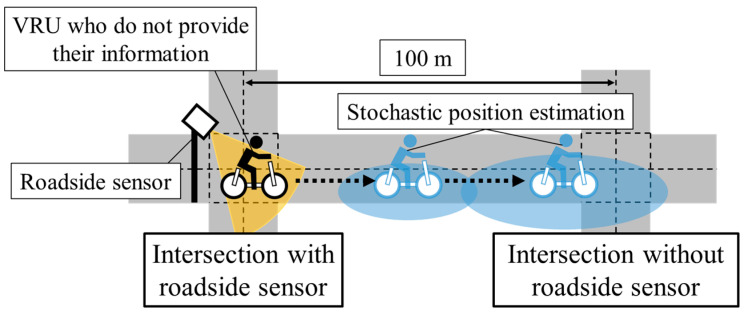
Conceptual schematic of the assumed situation in this study.

Considering the above challenge, in this study, we assume a situation shown in Figure 3. Because we assume a traffic environment with sparsely placed roadside sensors, there is a roadside sensor only at the left intersection. In such a traffic environment, a cyclist moves from the left intersection to the right one. Here, we assume 100 m for the distance, which is a relatively long distance between intersections in Japan, though the distance between intersections varies in each town. For the prevention of crossing collisions at the second intersection, it is necessary to estimate the position using information which is observed previously at the first intersection. Here, in addition to the position estimation, it is also important to estimate uncertainty using the stochastic position estimation method. Under the assumed situation in this study, the estimation uncertainty by the usual stochastic position estimation method increases due to the lack of real-time information. Because small estimation uncertainties are desirable for safety actions by ADASs [10], compensation for the lack of real-time information is necessary. Although real-time information is not obtained in the situation, it is possible to roughly estimate the movement of cyclists from general characteristics or accumulated data.

**Figure 4:**
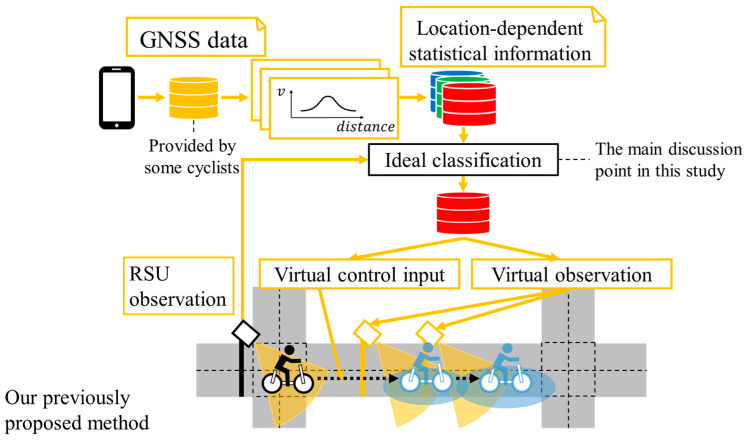
Conceptual schematic of our previously proposed method.

Based on such backgrounds and the existing studies related to virtual observation (VO) [11,12,13,14,15,16], we proposed methods to generate VOs and virtual control input (VCI) from Literature-Based Statistical Information (LBSI) [17], and from accumulated Global Navigation Satellite System (GNSS) data [18] in our previous studies. Figure 4 shows the conceptual schematic of our previously proposed method [18]. In the method, we considered spatial changes in cyclists’ movement characteristics depending on locations, such as deceleration around intersections, by using accumulated GNSS data. In particular, we constructed Location-Dependent Statistical Information (LDSI) from the accumulated data and utilized LDSI for position estimation by combining it with the observation information of Roadside Unit (RSU). Additionally, we considered a variety of movement characteristics of individuals by constructing multiple LDSIs based on clustering results of accumulated data. However, the above method assumed an ideal classification method as shown in Figure 4 and did not consider classification uncertainty. Thus, the performance of the position estimation method decreases in the actual environment where misclassification is inevitable to a certain degree. Specifically, it may use inappropriate parameters of the movement characteristics for cyclists’ position estimation when cyclists behave similarly inside the observation range of the RSU and differently outside the observation range. The utilization of the inappropriate parameters of the movement characteristics results in inappropriate position estimation and decreases its performance. Therefore, to estimate cyclists’ position appropriately, consideration of classification uncertainty is necessary.

Given the above motivations, this study proposes a method that considers classification uncertainty and estimates cyclists’ position using classification uncertainty. In addition, because the previously proposed method was evaluated only by simulations, we evaluate the position estimation method using LDSI with actual roadside sensor observations in this study. In summary, the main contributions of this study are as follows:

- Proposal of an LDSI-based position estimation method that can consider classification uncertainty.
- Evaluation of the proposed method in the actual environment.

The remainder of this paper is organized as follows. Section 2 presents the existing studies related to the position estimation of traffic participants. Section 3 explains the details of the proposed method. Section 4 describes the evaluation of the proposed method by simulations and experiments. Section 5 summarizes the conclusions.

## 2. Related Works

This study aims to develop a position estimation method that focuses on variations in cyclists’ movement characteristics. Thus, Section 2.1 organizes research on variations in the characteristics. In addition, cyclists’ movement characteristics are considered to be different due to their attributes. Therefore, Section 2.2 organizes the estimation method of traffic participants’ attributes. Finally, Section 2.3 organizes the position estimation methods that consider mobility diversity based on accumulated data.

### 2.1. Variety of Cyclists’ Movement Characteristics

The consideration of the movement characteristics of cyclists is important not only for traffic safety but also for smooth traffic. Thus, various studies related to cyclists’ movement characteristics have been conducted. Eriksson et al. [19] installed RSUs at several points and analyzed cyclists’ velocity at each point. As a result, they found that cyclists’ velocity is affected by slopes, intersections, and the time of day. Clarry et al. [20] accumulated GNSS data of cyclists and analyzed the factors that affect cyclists’ velocity. They found that bicycle infrastructures, slopes, distance from intersections, day of week, and time of day affect cyclists’ velocity. Twisk et al. [21] collected data with accelerometers and GNSS and analyzed cyclists’ velocity and acceleration/deceleration. As for the velocity, they found that the average velocity in rural areas is higher than that in urban areas. In addition, they found that males travel faster than females. Yan et al. [22] collected GNSS data of cyclists and analyzed the variations in speed by individual and location. From the analysis, they found that cyclists’ preferences, time of day, slopes, intersections, and land use type (e.g., transport, industry, nature area) affect their speed.

In addition to the research related to the cyclists’ velocity, some research focused on their behavior. Bernhoft et al. [23] conducted a questionnaire survey of older cyclists and younger cyclists and analyzed their preferences and their behavior. The results showed that older cyclists tend to obey the law. O’Hern et al. [24] investigated the relationship between cyclists’ behavior and their personality traits using a cycling behavior questionnaire. The results showed a positive association between extroversion and both errors and violations. Oehl et al. [25] invented the “Cycling Anger Scale” and investigated the factors related to cyclists’ anger and their behavior. The results showed that anger scores are related to cyclists’ gender and their cycling years. They also found positive correlations between cycling anger and risky cycling behavior.

The above studies show that various factors affect cyclists’ velocity and their behavior. Thus, to estimate the cyclists’ movement appropriately, consideration of their diverse movement characteristics is necessary. In particular, because many studies have demonstrated that the movement characteristics differ by individuals and location, consideration of these factors is important.

### 2.2. Estimation of Traffic Participants’ Attributes

Much research related to the estimation of traffic participants’ attributes has been conducted in the field of computer vision. Although the primary focus of research in the field is not on cyclists but on pedestrians, who are a kind of vulnerable road user, discussions of attribute estimation of pedestrians are also useful for those of cyclists. Thus, we organize the following research including pedestrian-related research in this section. Li et al. [26] estimated pedestrians’ age, gender, and clothing from images by using a deep learning-based method. They also proposed another attribute estimation method that considered pedestrians’ poses and improved the performance of the estimation [27]. Liu et al. [28] proposed an attribute estimation method that could consider multiscale features in images by using “multidirectional attention modules.” Zeng et al. [29] proposed an estimation method that estimated multiple attributes using multiple networks with a soft parameter shared structure.

In addition to the above attribute estimation method which uses images, some studies investigated methods using radars and Light Detection and Rangings (LiDARs). Horn et al. [30] used radar observation to estimate pedestrians’ height in addition to their motion (e.g., walk, run, jump). Shen et al. [31] used LiDAR observation for gait recognition, which is used for the identification of individuals.

As mentioned above, most of the attribute estimation methods use images. However, because this study focuses on the community roads, the use of cameras is partly limited due to privacy concerns. Additionally, considering the limited number of attribute estimation methods using radars or LiDARs, we can consider that it is hard to estimate traffic participants’ attributes, such as gender and age, using such sensors. Therefore, we classify the attributes of cyclists based on their movement observed inside the observation range of RSUs. In other words, we focus on the cyclists’ movement characteristics rather than on their gender or age, and do not use the attributes estimation method explained above.

### 2.3. Data-Driven Position Estimation Methods Considering Mobility Diversity

As mentioned in Section 2.1, cyclists’ movement characteristics differ by location and individual. Thus, to estimate cyclists’ position appropriately, consideration of the diversity in the movement characteristics based on accumulated data is effective. In general, there are two approaches in data-driven position estimation methods: machine learning (ML)-based methods and physical-model-based methods. ML-based methods do not clearly define motion models. In contrast, physical-model-based methods clearly define the models and adjust the parameters based on the accumulated data.

As an example of ML-based methods, Chai et al. [32] predicted vehicles’ future trajectories multimodally, considering drivers’ intentions (e.g., lane changes, slowing down). Abdelraouf et al. [33] personalized the pre-trained estimation model by transfer learning and estimated the trajectories of vehicles appropriately. Li et al. [34] classified vehicle driving style and trained the trajectory prediction network by using the classification results. Chen et al. [35] proposed a trajectory prediction method that could consider personalized motion patterns by learning the latent distribution of different motion patterns.

As an example of physical-model-based methods, Dyckmanns et al. [36] proposed a trajectory estimation method using the Interacting Multiple Model (IMM), which uses prior knowledge such as traffic volume to adapt the transition matrix. Jeong et al. [37] clustered accumulated driving data at a certain intersection and defined multiple extended Kalman filter (EKF) models for each cluster. Subsequently, they estimated vehicle motion using IMM. Yi et al. [38] clustered accumulated trajectories at a certain intersection and predicted drivers’ intention by training the classification and regression tree algorithm.

Because the former methods using ML do not define motion models, such methods cannot use the observation information in real-time from sensors that is not used in the data accumulation. Therefore, we use the latter one, which defines motion models. Against the above background, we proposed a method that utilizes VO adjusted by LBSI for KF in our previous study [17]. VO compensated for the lack of real-time information and mitigated the increase in the estimation uncertainty. However, because LBSI, which is independent of location, is rough information, it cannot consider movement characteristics that depend on a certain location, such as deceleration around intersections. To overcome such limitations, we proposed a method that analyzed the cyclists’ movement characteristics from accumulated GNSS data and utilized the analyzed results as VOs and VCI of the EKF in our other previous study [18]. To be more precise, we analyzed the movement characteristics along roads from accumulated GNSS data and constructed LDSI. Additionally, to consider a variety of movement characteristics of individuals, we constructed multiple LDSIs based on clustering of accumulated data. As a result of the experiment for data accumulation and the numerical simulation based on the data, our previously proposed method estimated the cyclists’ position more appropriately than the conventional method.

However, our previously proposed method assumes an ideal classification of cyclists. Thus, it cannot consider classification uncertainty in the actual environment where misclassification is inevitable to a certain degree. Therefore, we propose a position estimation method that utilizes LDSI with consideration of classification uncertainty in this study. Table 1 summarizes the above discussion. Although ML-based methods and usual physical-model-based methods can consider classification uncertainties, these methods assume that the real-time information of targets is always available. Thus, the key feature of the proposed method in this study is that it simultaneously compensates for the lack of the real-time information and considers classification uncertainty.

## 3. Methods

### 3.1. Investigation Situation

**Figure 5:**
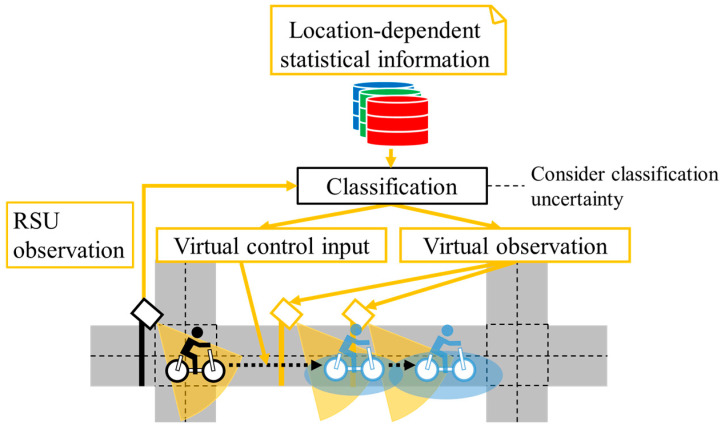
Conceptual schematic of the investigation situation in real-time estimation.

As already mentioned in Section 1, we estimate cyclists’ position using real-time observation information from sparsely placed roadside sensors and VO information from LDSI. Figure 5 shows the conceptual schematic of the investigation situation. Here, because we assume that cyclists do not provide GNSS data, there is no real-time information available outside the observation range of the real RSU. However, LDSI is constructed in advance from accumulated GNSS data provided by those who agree to provide their data. Thus, LDSI is available even in the assumed situation where there is no real-time information available. Additionally, because GNSS data is used for LDSI construction, LDSI is available outside the observation range of real roadside sensors. In summary, by compensating for a lack of real-time information with the VO, we continue to estimate cyclists’ position even when they exit the observation range of the real roadside sensor.

### 3.2. Conceptual Design of the Proposed System

#### 3.2.1. Overview of Proposed Method

**Figure 6:**
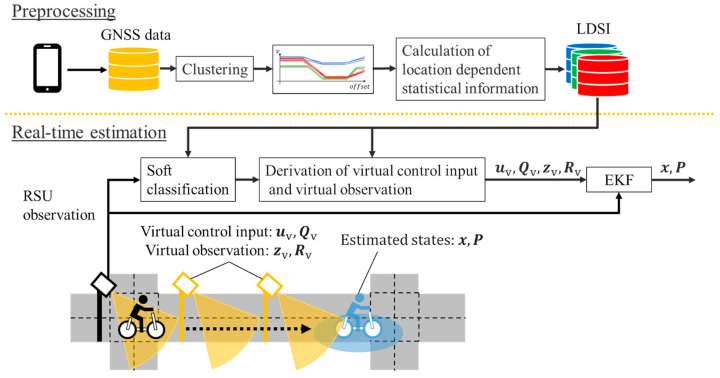
Overview of the proposed method that includes the preprocessing part and the real-time estimation part. Red, green, and blue indicate the corresponding clusters.

In this section, we first explain the overview of the proposed method including the contribution of the previous study [18]. Subsequently, we explain the main discussion point of this study. Figure 6 shows the overview of the proposed method that includes the preprocessing part and the real-time estimation part. In the first part, we construct LDSI from the clustering result of accumulated GNSS data. In the second part, an observed cyclist by the roadside sensor is classified into the predefined clusters in LDSI. After that, the VO and the VCI are derived from the classification results and LDSI. Finally, cyclists’ position is estimated by EKF using the observation information from the roadside sensor, the VO, and the VCI.

**Figure 7:**
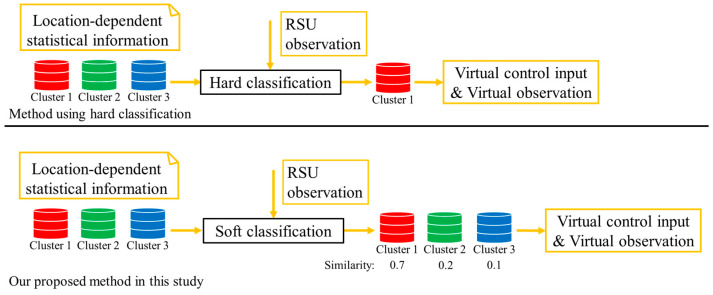
Conceptual schematic of the comparison between the proposed method in our previous study and the proposed method in this study.

The preprocessing part of the above method is already established in our previous study [18]. In addition, our previous study also established a derivation method of the VO and the VCI on the assumption that the cyclists’ cluster is ideally identifiable. However, in the real situation, the cyclists’ cluster is hardly identifiable. To be more precise, because LDSI is constructed without explicitly distinguishing the movement characteristics inside and outside the roadside sensor’s observation range, accurate classification is practically hard in the assumed situation where the movement characteristics outside the observation range cannot be observed. In other words, multiple clusters are confused in the classification, and the classification involves uncertainty in the real situation where the observation range is limited. Thus, the main objective of this study is to propose a position estimation method that considers such classification uncertainties. In this study, to consider classification uncertainty, we utilize a soft classification method and utilize its outputs as weight in the calculation of the VO and the VCI. Soft classification methods output the probability or confidence score of the target belonging to each class. Figure 7 shows the conceptual schematic of the comparison between the derivation process of VO and VCI using hard classification, and those in the proposed method. Here, hard classification is a classification method that discretely classifies objects into one class.

#### 3.2.2. Overview of LDSI

This section explains the overview of LDSI which was proposed in our previous study [18] and also utilized in this study. LDSI is statistical information related to cyclists’ movement characteristics at each location. In the construction of LDSI, the average and the standard deviation of the movement feature values, such as velocity and acceleration, are derived first. Such values are derived for each cluster of GNSS data. Subsequently, the derived values are associated with the map system to construct LDSI.

**Figure 8:**
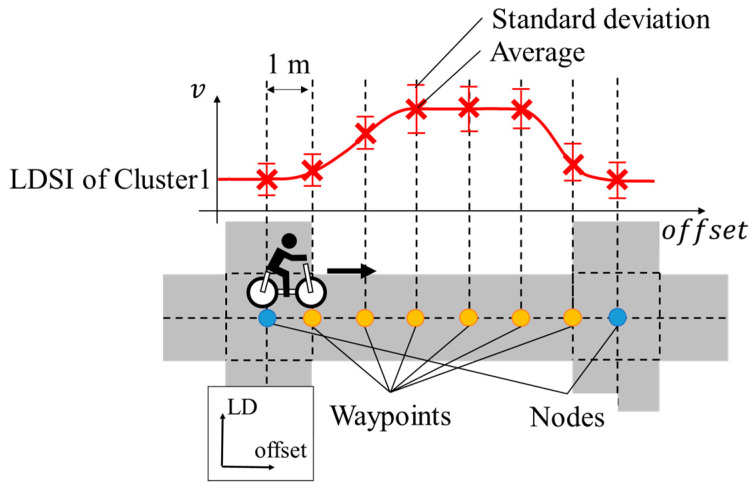
Conceptual schematic of a map system used in this study.

In this study, a relatively simple map system that consists of nodes and links is assumed. Nodes correspond to intersections, and links correspond to roads between intersections in the real world. Figure 8 shows the conceptual schematic of such a map system. As shown in Figure 8, we set the coordinate system along the road. Here, similar to our previous study related to the digital map system [39], we call the distance along the road “offset” and the distance perpendicular to the road “LD” (Lateral Deviation). LDSI is associated with the waypoints placed at a certain interval along the offset direction. In this study, we assume the interval of the waypoints considering the positional resolution of changes in cyclists’ movement characteristics. Because 0.1 m is too small an interval and 10 m is too large an interval for movement characteristic analysis, we assume the interval of waypoints as 1 m.

In our previous study [18], we utilized the VO and the VCI derived from LDSI when real-time information was not available. The VO and the VCI compensate for the lack of real observation and enable appropriate position estimation. In this study, we utilize the velocity and the directional angle as the VO, and the acceleration and the yawing rate as the VCI, similar to our previous study [18]. In the calculation of the VO and the VCI, we first derived intermediate values from LDSI, the cyclists’ cluster, and the estimated cyclists’ position. Then, we derived the VO, VCI, and their uncertainty from the corresponding intermediate values by considering effect of VO and VCI.

### 3.3. Definition of Variables Used in the Extended Kalman Filter

The formulas of the EKF are well known, and the formulas and the parameters of the EKF in this study are the same as those in our previous study [18]. Thus, this section explains only the definition of the state variables and control inputs used in this study for understanding the following sections.

In the EKF for cyclists’ movement estimation, the planar positions, the velocity, and the directional angle are often expressed as state variables. Here, because cyclists do not move laterally, the velocity and the directional angle are utilized. On the other hand, the acceleration and the yawing rate are often employed as control inputs. Similar to the general cases, in this study, the state variable x, the estimation covariance matrix P, and the control input u are expressed as follows:

(1) $$x={\left(\begin{array}{cccc} x & y & \theta & v \end{array}\right)}^{T}$$

(2) $$P=\left(\begin{array}{cccc} \sigma_{x}^{2} & \sigma_{xy} & \sigma_{x\theta } & \sigma_{xv}\\ \sigma_{xy} & \sigma_{y}^{2} & \sigma_{y\theta } & \sigma_{yv}\\ \sigma_{x\theta } & \sigma_{y\theta } & \sigma_{\theta }^{2} & \sigma_{\theta v}\\ \sigma_{xv} & \sigma_{yv} & \sigma_{\theta v} & \sigma_{v}^{2} \end{array}\right)$$

(3) $$u={\left(\begin{array}{cc} \omega & a \end{array}\right)}^{T}$$

where x and y denote the planar positions in the Cartesian coordinate system representing the East and North directions, θ denotes the directional angle, v denotes the velocity, ω denotes the yawing rate, and a denotes the acceleration. In addition, the process noise matrix Q is expressed as follows:

(4) $$Q=\left(\begin{array}{cc} \sigma_{\omega }^{2} & 0\\ 0 & \sigma_{a}^{2} \end{array}\right)$$

Here, 0 is associated with u, and the parameters derived from general movement characteristics are associated with Q when the control input is not available. As for the observation ***z*** and the measurement noise matrix R, because these variables depend on the characteristics of sensors, they are explained in Section 4. Using Equations (1)–(4), the transition function, and EKF formulas, the movement states of cyclists are estimated. The details of the formulas used in this process are shown in our previous study [18].

### 3.4. Cluster Estimation Method

#### 3.4.1. Conceptual Design of Cluster Estimation Method

**Figure 9:**
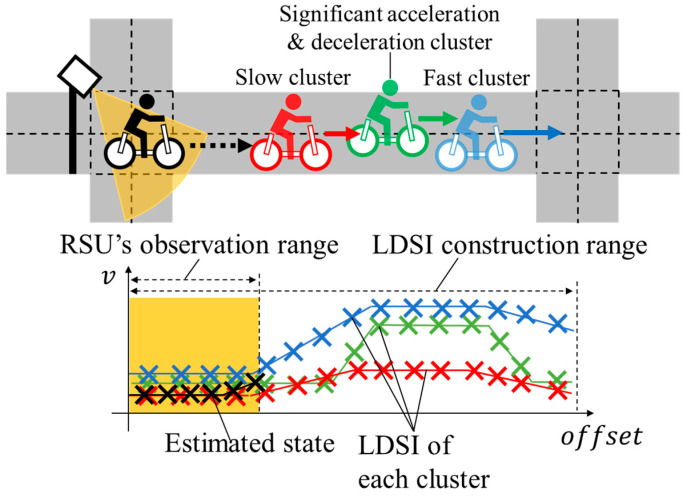
Conceptual schematic of the classification in this study.

As mentioned in Section 3.2, we utilize a soft classification method to consider the classification uncertainty in the following process of the position estimation. Because no information is available outside the observation range of the roadside sensor, only the estimated states by the EKF inside the observation range are utilized for the classification. Additionally, for the same reason, the classification is conducted only once after the cyclists exit the observation range. Figure 9 shows the conceptual schematic of the classification. As shown in Figure 9, the observation range of the roadside sensor does not cover the whole range used for the LDSI construction. In such a situation, each cluster of cyclists may have similar movement characteristics inside the observation range although each cluster of cyclists has different movement characteristics outside the observation range. Thus, the soft classification method which can consider classification uncertainty is necessary for appropriate position estimation.

There are various methods related to soft classification, such as multiple logistic regression. However, most of the soft classification methods require a learning process or parameter adjustment for each situation and cannot utilize observations that are not included in the accumulated data. Related to this, observations that are not available every time but available occasionally, such as the observation information from the connected vehicles, are useful and it is desirable to handle such information. However, the above soft classification methods cannot utilize such information. Therefore, we utilize a classical approach for soft classification. To be more precise, we calculate the similarity between cyclists’ movement characteristics and the LDSI at each waypoint and utilize the calculated similarity for soft classification.

#### 3.4.2. Implementation of Cluster Estimation Method

The process of the soft classification method used in this study is as follows:

1. Interpolation of time-series estimated states to the waypoint.
2. Calculation of similarity between the estimated states and LDSI at each waypoint.
3. Calculation of whole similarity between estimated states and LDSI of each cluster.

In the interpolation of estimated states to the waypoint, we first transform the estimated cyclists’ position, which is estimated in the Cartesian coordinate, to the offset-LD coordinate. The transformation equations are expressed as follows:

(5) $$M_{\psi }=\left(\begin{array}{cc} \cos\psi & -\sin\psi \\ \sin\psi & \cos\psi \end{array}\right)$$

(6) $$\left(\begin{array}{c} o\\ l \end{array}\right)=M_{\psi }^{-1}\left(\begin{array}{c} x-x_{C}\\ y-y_{C} \end{array}\right)+\left(\begin{array}{c} o_{C}\\ l_{C} \end{array}\right)$$

where ψ denotes the angle from the *x*-axis to the offset-axis with counterclockwise rotation as positive, $M_{\psi }$ denotes the rotation matrix, $x_{C}$ and $y_{C}$ denote the planar positions of the closest waypoint from the current position, o and l denote the position in the offset-LD coordinate system, and $o_{C}$ and $l_{C}$ denote the offset and LD of the closest waypoint from the current position, respectively. The transformation of the coordinate system regarding the estimated covariance matrix is expressed as follows:

(7) $$\left(\begin{array}{cc} \sigma_{o}^{2} & \sigma_{ol}\\ \sigma_{ol} & \sigma_{l}^{2} \end{array}\right)=M_{\psi }^{-1}\left(\begin{array}{cc} \sigma_{x}^{2} & \sigma_{xy}\\ \sigma_{xy} & \sigma_{y}^{2} \end{array}\right)M_{\psi }$$

where $\sigma_{o}$ and $\sigma_{l}$ denote the estimation uncertainties of the offset and LD positions, respectively, and $\sigma_{ol}$ denotes the covariance of the offset and LD positions. After the coordinate transformation, we spatially resample the estimated states by EKF, which are obtained in the time series, at each waypoint.

In the similarity calculation at each waypoint, we consider the uncertainty of the estimated states and the distribution of LDSI. Because the cyclists’ movement states are estimated by EKF, the uncertainties of the movement states are estimated simultaneously. On the contrary, the LDSI provides information on the distribution of cyclists’ movement characteristics and consists of the average and the standard deviation. To appropriately estimate the cyclists’ cluster in LDSI, the above uncertainty and the above distribution should be considered.

**Figure 10:**
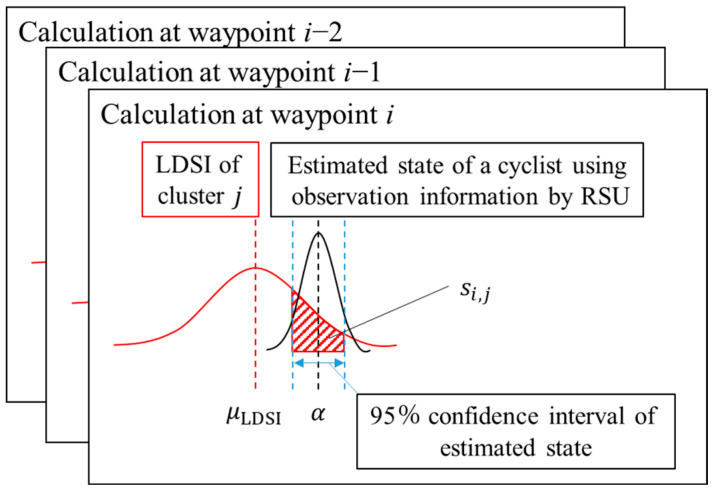
Conceptual schematic of the likelihood calculation at each waypoint.

Considering the above points, we evaluate the likelihood that the observed cyclist belongs to each cluster. To evaluate the likelihood, we focus on the overlap of LDSI’s distribution and the estimated distribution of movement states in real-time. To be more precise, we calculate the integral of LDSI’s distribution over the 95% confidence interval (95%CI) of the states estimated in real-time. Figure 10 shows the conceptual schematic of the likelihood calculation at each waypoint. The calculated likelihood s is expressed as follows:

(8) $$s_{i,j}=\int_{\alpha_{95\_min}}^{\alpha_{95\_max}}\frac{1}{\sqrt{2\pi \sigma_{i,j,\;LDSI}^{2}}}\exp\left\{-\frac{{\left[\alpha -\mu_{i,j,\;LDSI}\right]}^{2}}{2\sigma_{i,\;j,LDSI}^{2}}\right\}d\alpha$$

where α denotes a state, i denotes the index of a waypoint, j denotes the index of a cluster in LDSI, $\sigma_{i,j,\;LDSI}$ denotes the standard deviation of LDSI, $\mu_{i,j,\;LDSI}$ denotes the average of LDSI, and $\alpha_{95\_max}$ and $\alpha_{95\_min}$ denote the upper limit and the lower limit of the 95%CI, respectively. In the above equation, the effect of the distribution of LDSI in each cluster to likelihood $s_{i,j}$ and that of the distribution of the estimated state are different. To explain the above difference, we organize the effect of the standard deviation of LDSI and that of the estimated state. As for the distribution of LDSI, when the mean of LDSI and that of the estimated state are close, likelihood $s_{i,j}$ increases as the standard deviation of LDSI decreases. In contrast, when the mean of LDSI and that of the estimated state differ, likelihood $s_{i,j}$ decreases as the standard deviation of LDSI decreases. On the other hand, as for the distribution of the estimated state, likelihood $s_{i,j}$ tends to become large when the standard deviation of the estimated state is large and 95%CI of the estimated state is large. Here, although the standard deviation of the LDSI affects only the likelihood $s_{i,j}$ to the corresponding cluster, the standard deviation of the estimated state affects that of all clusters. Thus, in this case, likelihood $s_{i,j}$ of all clusters tends to become large and the difference between each cluster is hardly considered. Conversely, when the 95%CI of the estimated state is small, the likelihood tends to differ across all clusters. Thus, the difference between each cluster is well considered.

In the calculation of whole similarity between estimated states and LDSI of each cluster, we calculate the summarizing index from the calculated likelihood at each waypoint. To be more precise, we calculate the average of the likelihood at each waypoint and utilize it as the summarizing index for the similarity. The summarizing index of similarity $S_{j}$ is expressed as follows:

(9) $$S_{j}=\frac{1}{n}\sum_{i}^{n}s_{i,j}$$

where n denotes the number of the waypoints. Subsequently, to utilize the summarizing index of each cluster in LDSI in the following estimation, we normalize these indices by the following formula:

(10) $$S_{j}^{\prime }=\frac{S_{j}}{\sum_{j}S_{j}}$$

The above classification process assumes classification based on a one-dimensional movement characteristic. The above classification process can be applied to multi-dimensional movement characteristics by extending Equation (8). Here, the planar positions x and y, the directional angle θ, and the velocity v, which are the variables estimated by EKF, are considered. However, because the planar positions are utilized to refer to the LDSI, the planar positions cannot be utilized for classification. Additionally, because this study assumes cyclists’ movement on straight links between intersections, the directional angle basically does not differ. Therefore, we utilize only the velocity for the classification and we do not utilize the planar positions and the directional angle for the classification.

### 3.5. VO and VCI Considering Cluster Estimation

#### 3.5.1. VO and VCI in Our Previous Study

In this study, the basic formulas used in the calculation of the VO and the VCIs are the same as those in our previous study [18]. Thus, this section outlines the variables of the VO and the VCI based on our previous study. The details of the calculation are shown in Appendix A.

As already mentioned in Section 3.2.2, the velocity and directional angle are utilized as the VO, and the acceleration and the yawing rate are utilized as the VCI. Here, the intermediate values $\tilde{\mu }$ and $\tilde{\sigma }$ are calculated first using LDSI, the cyclists’ cluster, and the estimated cyclists’ position. The calculation process of the intermediate values is explained in our previous study [18]. The VO and the VCI are derived from the intermediate values $\tilde{\mu }$ with corresponding subscripts.

The VCI $u_{v}$ and the VO $z_{v}$ are expressed as follows:

(11) $$u_{v}\left(t_{k-1}\right)={\left(\begin{array}{cc} {\tilde{\mu }}_{\omega }\left(t_{k-1}\right) & {\tilde{\mu }}_{a}\left(t_{k-1}\right) \end{array}\right)}^{T}$$

(12) $$z_{v}\left(t_{k}\right)={\left(\begin{array}{cc} {\tilde{\mu }}_{\theta }\left(t_{k}\right) & {\tilde{\mu }}_{v}\left(t_{k}\right) \end{array}\right)}^{T}$$

(13) $$H_{v}=\left(\begin{array}{cccc} 0 & 0 & 1 & 0\\ 0 & 0 & 0 & 1 \end{array}\right)$$

where $t_{k}$ denotes discretized time, ${\tilde{\mu }}_{\omega }$, ${\tilde{\mu }}_{a}$, ${\tilde{\mu }}_{\theta }$, and ${\tilde{\mu }}_{v}$ denote the intermediate values of yawing rate, acceleration, directional angle, and velocity, respectively.

As for the uncertainty of VCI $Q_{v}$ and the uncertainty of VO $R_{v}$, they are derived from intermediate values $\tilde{\sigma }$ with corresponding subscripts, and safety factors ${sf}_{Q}$ and ${sf}_{R}$, respectively. The derivations of the uncertainty of VCI $Q_{v}$ and the uncertainty of VO $R_{v}$ are as follows:

(14) $$Q_{v}\left(t_{k-1}\right)=\left(\begin{array}{cc} {{\tilde{\sigma }}_{\omega }^{\prime }}^{2}\left(t_{k-1}\right) & 0\\ 0 & {{\tilde{\sigma }}_{a}^{\prime }}^{2}\left(t_{k-1}\right) \end{array}\right)\;\;\;\;\;\;\;\;\;\;\;\;\;\;=\left(\begin{array}{cc} {\left(1+sf_{Q}\right)}^{2}{\tilde{\sigma }}_{\omega }^{2}\left(t_{k-1}\right) & 0\\ 0 & {\left(1+sf_{Q}\right)}^{2}{\tilde{\sigma }}_{a}^{2}\left(t_{k-1}\right) \end{array}\right)$$

(15) $$R_{v}\left(t_{k}\right)=g\left({{\tilde{\sigma }}_{\theta }^{\prime }}^{2}\left(t_{k}\right),{{\tilde{\sigma }}_{v}^{\prime }}^{2}\left(t_{k}\right),{\tilde{\sigma }}_{\omega }^{\prime }\left(t_{k-1}\right),{\tilde{\sigma }}_{a}^{\prime }\left(t_{k-1}\right),∆t\right)\;\;\;\;\;\;\;\;\;\;\;\;\;\;\;\;=g\left({\left(1+{sf}_{R}\right)}^{2}{\tilde{\sigma }}_{\theta }^{2}\left(t_{k}\right),{\left(1+{sf}_{R}\right)}^{2}{\tilde{\sigma }}_{v}^{2}\left(t_{k}\right),{\tilde{\sigma }}_{\omega }^{\prime }\left(t_{k-1}\right),{\tilde{\sigma }}_{a}^{\prime }\left(t_{k-1}\right),∆t\right)$$

where g denotes the formula to calculate the measurement noise matrix of VO, ∆t denotes the observation interval, ${\tilde{\sigma }}^{\prime }$ with subscripts denotes the intermediate values after the correction, and $\tilde{\sigma }$ with subscripts denotes the intermediate values before the correction. The details of g are explained in Appendix A. Here, the safety factors ${sf}_{Q}$ and ${sf}_{R}$ are employed to stably estimate the position of cyclists whose movement states contain relatively outliers in the cluster. In this study, similar to our previous study, we assigned 0.3 to ${sf}_{Q}$ and ${sf}_{R}$ from the preliminary investigation.

As already mentioned, the above calculation process, which was utilized in our previous study, cannot consider classification uncertainty. To consider the uncertainty, we propose a position estimation method using soft classification results. In the following paragraph, we call the method a soft classification (SC)-based method. The details of the SC-based method are explained in Section 3.5.3.

#### 3.5.2. Calculation of the Intermediate Values in the Conventional Method

In this study, we employ three conventional methods as a baseline for comparison: position estimation using LBSI, position estimation using the ground truth (GT) cluster, and position estimation using hard classification (HC). In the following paragraph, we call the above methods the LBSI-based method, the GT cluster-based method, and the HC-based method, respectively. In the above conventional methods, derivation of the intermediate values $\tilde{\mu }$ and ${\tilde{\sigma }}^{\prime }$ are different from each other. The following process after the calculation of the intermediate values are same in the three methods.

- LBSI-based method

The LBSI-based method is the method proposed in our previous study [17]. LBSI provides general statistical information on cyclists’ movement characteristics but does not provide information for a specific location. Additionally, LBSI does not provide information on acceleration and yawing rate, which heavily depend on the location. Thus, only the VO is utilized in this method, and the VCI is not utilized.

Here, because the same method using LBSI was utilized as the conventional method in our previous study [18], we utilize the same intermediate values for $\tilde{\mu }$ and ${\tilde{\sigma }}^{\prime }$. To be more precise, we assume the intermediate values for VO calculation related to velocity are as follows:

(16) $${\tilde{\mu }}_{v}=4.2\;m/s$$

(17) $${\tilde{\sigma }}_{v}^{\prime }=1.4\;m/s$$

In addition, the intermediate values for VO calculation related to the directional angle are as follows:

(18) $${\tilde{\mu }}_{\theta }=\theta_{link}$$

(19) $${\tilde{\sigma }}_{\theta }^{\prime }=0.13\;rad$$

where $\theta_{link}$ denotes the directional angle of the road.

- GT cluster-based method

The GT cluster-based method is the method proposed in our previous study [18]. This method utilizes the formulas explained in Section 3.5.1. The intermediate values $\tilde{\mu }$ and ${\tilde{\sigma }}^{\prime }$ utilized in the VO and the VCI are calculated from the LDSI of the GT cluster of cyclists. Because the GT cluster of cyclists is not identifiable in the real world, this method is used only in the simulation evaluation for reference.

- HC-based method

**Figure 11:**
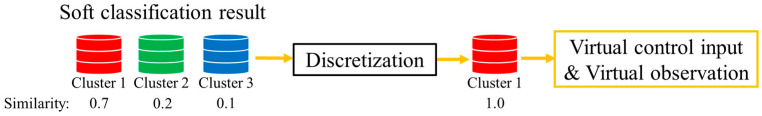
Conceptual schematic of the discrete classification used in this study.

The HC-based method is the method which does not consider classification uncertainty. Figure 11 shows the conceptual schematic of the discrete classification used in this study. Here, we utilize the calculated similarity between cyclists’ movement characteristics and LDSI of each cluster, which are explained in Section 3.4, for the hard classification. As shown in Figure 11, cyclists are discretely classified into the cluster with the maximum similarity. Then, the LDSI of the corresponding cluster is utilized in the calculation of the intermediate values $\tilde{\mu }$ and ${\tilde{\sigma }}^{\prime }$. The following process is the same as the method with the GT cluster.

#### 3.5.3. Calculation of the Intermediate Values in Proposed Method

In this study, we propose a position estimation method that considers classification uncertainty by using soft classification results. The proposed method merges VO and VCI by using soft classification results. In particular, we first derive the VO and the VCI from the LDSI of each cluster. Subsequently, VO and the VCI are merged using soft classification results. The merged VCI $u_{v,m}$ and the merged VO $z_{v,m}$ are expressed as follows:

(20) $$u_{v,m}\left(t_{k-1}\right)=\sum_{j}^{N}S_{j}^{\prime }u_{v,j}\left(t_{k-1}\right)$$

(21) $$z_{v,m}\left(t_{k}\right)=\sum_{j}^{n}S_{j}^{\prime }z_{v,j}\left(t_{k}\right)$$

where $S_{j}^{\prime }$ denotes the normalized similarity derived in the soft classification, and $u_{v,j}$ and $z_{v,j}$ denote the VCI and the VO calculated from *j*th cluster in LDSI, respectively.

As for the uncertainty of the merged VCI $Q_{v,m}$ and the uncertainty of the merged VO $R_{v,m}$ are expressed as follows:

(22) $$Q_{v,m}\left(t_{k-1}\right)=\left(\begin{array}{cc} {{\tilde{\sigma }}_{\omega ,m}^{\prime }}^{2}\left(t_{k-1}\right) & 0\\ 0 & {{\tilde{\sigma }}_{a,m}^{\prime }}^{2}\left(t_{k-1}\right) \end{array}\right)$$

(23) $${{\tilde{\sigma }}_{\omega ,m}^{\prime }}^{2}\left(t_{k-1}\right)={\left(1+{sf}_{Q}\right)}^{2}{\tilde{\sigma }}_{\omega ,m}^{2}\left(t_{k-1}\right)\;\;\;\;\;\;\;\;\;\;\;\;\;\;\;\;\;={\left(1+{sf}_{R}\right)}^{2}\sum_{j}^{N}S_{j}^{\prime }\left\{{\left[{\tilde{\mu }}_{\omega ,m}\left(t_{k-1}\right)-{\tilde{\mu }}_{\omega ,j}\left(t_{k-1}\right)\right]}^{2}+{\tilde{\sigma }}_{\omega ,j}^{2}\left(t_{k-1}\right)\right\}$$

(24) $${{\tilde{\sigma }}_{a,m}^{\prime }}^{2}\left(t_{k-1}\right)={\left(1+{sf}_{Q}\right)}^{2}{\tilde{\sigma }}_{a,m}^{2}\left(t_{k-1}\right)\;\;\;\;\;\;\;\;\;\;\;\;\;\;\;\;\;={\left(1+{sf}_{R}\right)}^{2}\sum_{j}^{N}S_{j}^{\prime }\left\{{\left[{\tilde{\mu }}_{a,m}\left(t_{k-1}\right)-{\tilde{\mu }}_{a,j}\left(t_{k-1}\right)\right]}^{2}+{\tilde{\sigma }}_{a,j}^{2}\left(t_{k-1}\right)\right\}$$

(25) $$R_{v,m}\left(t_{k}\right)=g\left({{\tilde{\sigma }}_{\theta ,m}^{\prime }}^{2}\left(t_{k}\right),{{\tilde{\sigma }}_{v,m}^{\prime }}^{2}\left(t_{k}\right),{\tilde{\sigma }}_{\omega ,m}^{\prime }\left(t_{k-1}\right),{\tilde{\sigma }}_{a,m}^{\prime }\left(t_{k-1}\right),∆t\right)$$

(26) $${{\tilde{\sigma }}_{\theta ,m}^{\prime }}^{2}\left(t_{k}\right)={\left(1+{sf}_{R}\right)}^{2}{\tilde{\sigma }}_{\theta ,m}^{2}\left(t_{k}\right)\;\;\;\;\;\;\;\;\;\;\;\;\;\;\;\;\;={\left(1+{sf}_{R}\right)}^{2}\sum_{j}^{N}S_{j}^{\prime }\left\{{\left[{\tilde{\mu }}_{\theta ,m}\left(t_{k}\right)-{\tilde{\mu }}_{\theta ,j}\left(t_{k}\right)\right]}^{2}+{\tilde{\sigma }}_{\theta ,j}^{2}\left(t_{k}\right)\right\}$$

(27) $${{\tilde{\sigma }}_{v,m}^{\prime }}^{2}\left(t_{k}\right)={\left(1+{sf}_{R}\right)}^{2}{\tilde{\sigma }}_{v,m}^{2}\left(t_{k}\right)\;\;\;\;\;\;\;\;\;\;\;\;\;\;\;\;\;={\left(1+{sf}_{R}\right)}^{2}\sum_{j}^{N}S_{j}^{\prime }\left\{{\left[{\tilde{\mu }}_{v,m}\left(t_{k}\right)-{\tilde{\mu }}_{v,j}\left(t_{k}\right)\right]}^{2}+{\tilde{\sigma }}_{v,j}^{2}\left(t_{k}\right)\right\}$$

where g denotes the formula to calculate measurement noise matrix of VO explained in Appendix A, ${\tilde{\sigma }}_{\omega ,m}^{\prime }$, ${\tilde{\sigma }}_{a,m}^{\prime }$, ${\tilde{\sigma }}_{\theta ,m}^{\prime }$, and ${\tilde{\sigma }}_{v,m}^{\prime }$ denote the corrected standard deviation of integrated yawing rate, acceleration, directional angle, and velocity, respectively, ${\tilde{\sigma }}_{\omega ,m}$, ${\tilde{\sigma }}_{a,m}$, ${\tilde{\sigma }}_{\theta ,m}$, and ${\tilde{\sigma }}_{v,m}$ denote the standard deviation of integrated yawing rate, acceleration, directional angle, and velocity before the correction, respectively, and ${\tilde{\sigma }}_{\omega ,j}$, ${\tilde{\sigma }}_{a,j}$, ${\tilde{\sigma }}_{\theta ,j}$, and ${\tilde{\sigma }}_{v,j}$ denote the intermediate values of yawing rate, acceleration, directional angle, and velocity of cluster j, respectively. The above equations are derived using a similar formula in the law of total variance. In the above equations, when the classification is accurate and the likelihood corresponding to a single cluster is large, $Q_{v,m}$ and $R_{v,m}$ are almost equivalent to those of the corresponding cluster. In contrast, when the classification is uncertain and the similarities to each cluster do not differ, $Q_{v,m}$ and $R_{v,m}$ tend to become large. To be more precise, when the LDSI inside the observation range of RSU are similar for multiple clusters and those outside the range are different, the classification is uncertain and $Q_{v,m}$ and $R_{v,m}$ become large. Due to such a feature, large $Q_{v,m}$ and $R_{v,m}$ can prevent over trust of VCI and VO when the classification is not accurate. The details of the overall calculation process including classification are organized in Appendix B.

**Figure 12:**
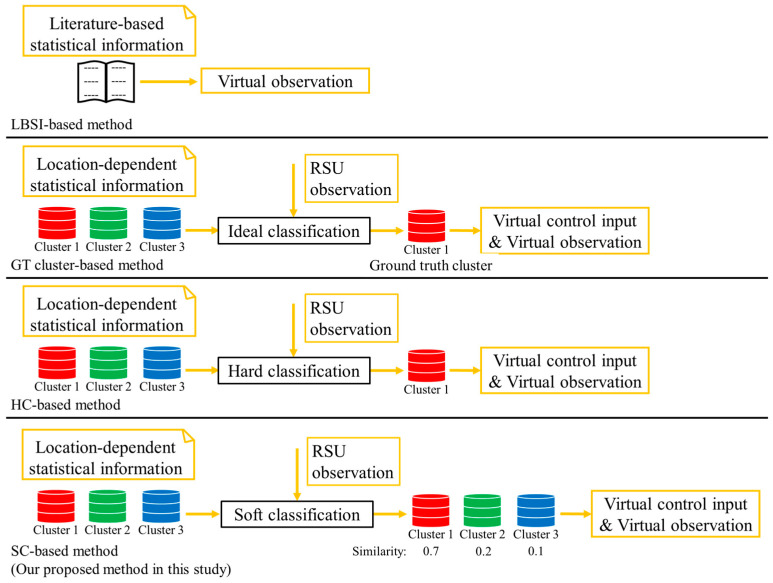
Conceptual schematic of the comparison between the proposed method in this study and the conventional methods for comparison.

From the discussion in the previous section and in this section, Figure 12 summarizes the conceptual schematic of the comparison between the proposed method in this study and the conventional methods for comparison.

## 4. Evaluation of the Proposed Methods

In this section, we evaluate the proposed methods in this study through simulation and experiment in the real world. In the simulation evaluation, we utilize the data used in the LDSI construction. In addition, we utilize the GT value as the roadside sensor’s observation. In summary, the simulation assumes ideal conditions where the cyclists’ movement characteristics are the same as those of LDSI, and ideal observation conditions.

On the contrary, in the experimental evaluation, the evaluation data is different from those used in the LDSI construction. Additionally, observation by an actual roadside sensor is utilized in the evaluation. In summary, the experimental evaluation assumes the condition where the cyclists’ movement characteristics are not guaranteed to be the same as those of LDSI, and utilizes the observation by an actual roadside sensor, which contains observation error.

### 4.1. Data for Simulation Experiment

In this section, we construct LDSI which is used in the following evaluation. In the simulation evaluation and the experiment evaluation, the same LDSI is used. The rest of Section 4.1 explains the data for LDSI construction and the constructed LDSI. Here, data for LDSI construction and the constructed LDSI are the same as our previous study [18].

#### 4.1.1. Experimental Setup for the Data Accumulation

In our previous study [18], we conducted an experiment to accumulate cyclists’ GNSS data in the Hongo campus of the University of Tokyo. Here, the characteristics of the road on the campus are similar to those of community roads: pedestrians and vehicles share the same area, and the visibility around intersections is poor. Thus, we consider the road on the campus as pseudo community roads. The experiment was conducted in accordance with the Declaration of Helsinki, and the protocol for this experiment was approved by the Research Ethics Committee, School of Engineering, The University of Tokyo (KE24-52) on 21 October 2024.

**Figure 13:**
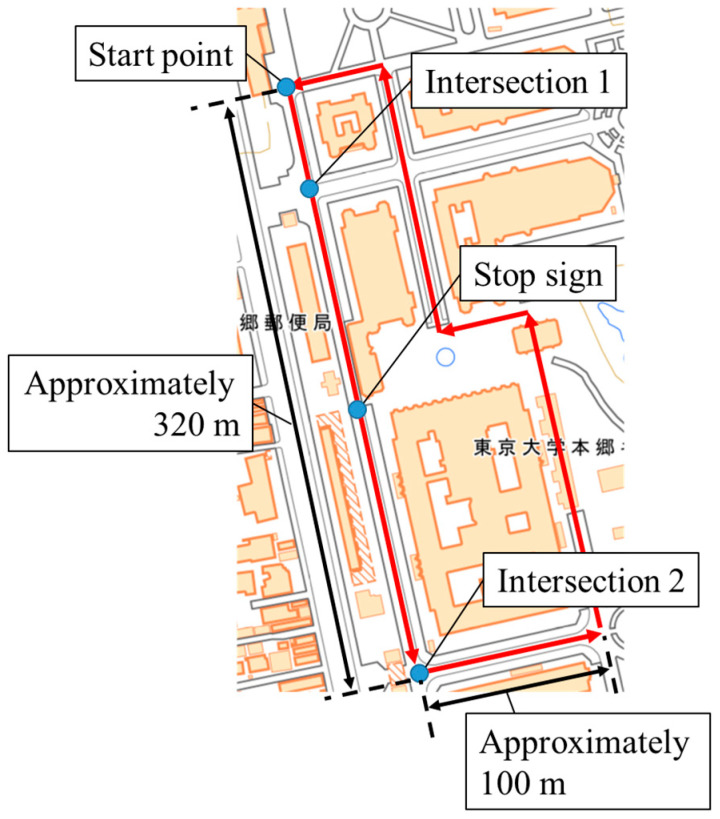
Map of the experiment course (created by editing the digital map [40]). The Chinese characters on the left side indicate a “post office”, and the Chinese characters on the right side indicate the “Hongo campus of the University of Tokyo”.

Ten experimental participants whose ages ranged from their twenties to their thirties were recruited for this experiment. We obtained written informed consent from the participants. Each participant cycled the experimental course, which is shown in Figure 13 [40], three times. The length of the experimental course was approximately 840 m. During the experiment, a smartphone was attached to the bicycle for accumulating GNSS data. Although 30 data was accumulated in total, one participant took the wrong course once. Thus, we excluded the data, and we finally obtained 29 valid data points in total. The summary of the data for LDSI construction is shown in Table 2.

#### 4.1.2. Constructed LDSI

**Figure 14:**
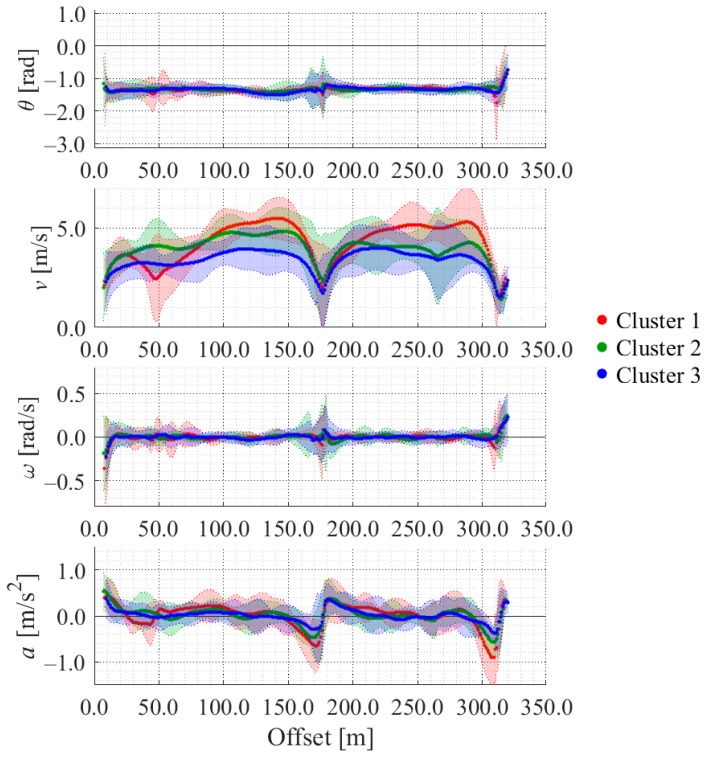
LDSI of each cluster.

Using the above data, we constructed LDSI using the method proposed in our previous study [18]. Figure 14 shows the constructed LDSI on a straight road from the start point to Intersection 2. The horizontal axis in the graphs indicates the offset, which is the distance from the start point along the road. In addition, lines of each color indicate the average of each cluster, and the areas of each color indicate the ±2σ range of each cluster. As shown in Figure 14, the movement characteristics of each cluster change by location. Additionally, the movement characteristics of each cluster differ from each other. As for velocity, Cluster 1 shows the large velocity change. Cluster 2 shows relatively high velocity. Cluster 3 shows relatively low velocity. In the following evaluation, we evaluate whether our proposed method can estimate cyclists’ position appropriately considering such differences in the movement characteristics. The constructed LDSI in this section is utilized in both the simulation evaluation and the experimental evaluation.

### 4.2. Evaluation Metrics

**Figure 15:**
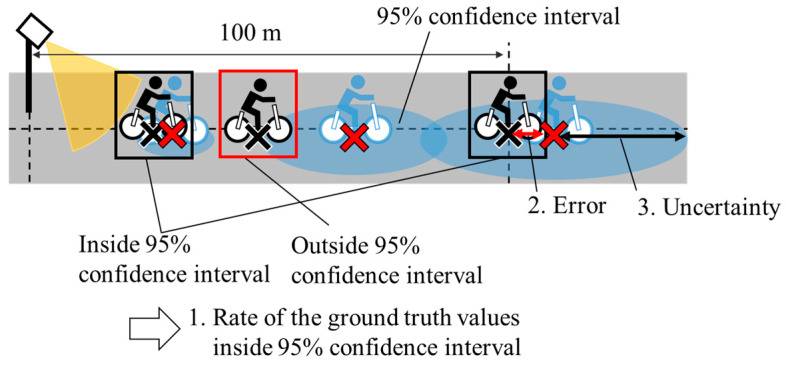
Conceptual schematic of the evaluation metrics. Black illustration and the black cross indicate the GT position of the cyclist. Blue illustration and the red cross indicate the estimated position of the cyclist.

In the evaluation of the stochastic estimation method, evaluations from the perspectives of both appropriateness and usefulness are necessary. Figure 15 shows the conceptual schematic of the evaluation metrics used in this study: 1. the rate of the GT within the 95%CI, 2. the estimation error, and 3. the uncertainty. The rates are calculated from the time when a cyclist exits the observation range of the roadside sensor to the time when a cyclist reaches a point 100 m ahead of the roadside sensor. As for the errors and the uncertainties for the evaluation, these values are calculated when a cyclist reaches a point 100 m ahead of the roadside sensor. Here, we employ 1σ for the evaluation metrics of uncertainty. Because a high rate of the GT values inside 95%CI and small estimation errors are necessary to prevent wrong estimation, these metrics are important for appropriateness evaluation. Additionally, because small uncertainties contribute to the appropriate behaviors of ADASs [10], small uncertainties are important from the perspective of usefulness. Furthermore, because small errors can prevent inappropriate behaviors of ADASs, small errors are also important from the perspective of usefulness. In summary, we use the metrics for the following purposes:

1. The rate of the GT values inside the 95%CI for appropriateness evaluation.
2. The estimated errors for both appropriateness and usefulness evaluations.
3. The estimated uncertainties for usefulness evaluation.

### 4.3. Simulation Evaluation

#### 4.3.1. Data for Simulation Evaluation

As already mentioned, the simulation evaluation utilizes the data used in the LDSI construction. Thus, we evaluate the performance of the proposed methods in ideal conditions where the cyclists’ movement characteristics are the same as those in the LDSI. Additionally, because the true clusters of cyclists are known in the data, which is used in the LDSI construction, we evaluate the performance of the soft classification in addition to the performance of the position estimation.

To utilize the data used in the LDSI construction, which is obtained at 1 Hz, for the simulation evaluation, we linearly interpolate the states estimated during the LDSI construction. To be more precise, we interpolate the estimated states at 1 Hz to the states at 10 Hz. In the following evaluation by the simulation, we utilize the interpolated values as the GT.

#### 4.3.2. Roadside Sensor Observation

In this study, we assume LiDAR as a roadside sensor. Although LiDAR cannot observe cyclists’ velocity and directional angle directly, those values calculated from the time-series position difference are useful for the movement states estimation. Thus, we utilize the position, the directional angle, and the velocity in the roadside sensor’s observation. Similar to our previous study [18], the formulas related to the roadside sensor’s observation are expressed as follows:

(28) $$\theta_{obs}\left(t\right)=atan2\left(y_{obs}\left(t\right)-y_{obs}\left(t-N∆t_{obs}\right),x_{obs}\left(t\right)-x_{obs}\left(t-N∆t_{obs}\right)\right)$$

(29) $$v_{obs}\left(t\right)=\frac{\sqrt{{\left[x_{obs}\left(t\right)-x_{obs}\left(t-N∆t_{obs}\right)\right]}^{2}+{\left[y_{obs}\left(t\right)-y_{obs}\left(t-N∆t_{obs}\right)\right]}^{2}}}{N∆t_{obs}}$$

(30) $$z={\left(\begin{array}{cccc} x_{obs} & y_{obs} & \theta_{obs} & v_{obs} \end{array}\right)}^{T}$$

(31) $$H=\left(\begin{array}{cccc} 1 & 0 & 0 & 0\\ 0 & 1 & 0 & 0\\ 0 & 0 & 1 & 0\\ 0 & 0 & 0 & 1 \end{array}\right)$$

(32) $$R=\left(\begin{array}{cccc} \sigma_{pos,obs}^{2} & 0 & 0 & 0\\ 0 & \sigma_{pos,obs}^{2} & 0 & 0\\ 0 & 0 & \sigma_{\theta ,obs}^{2} & 0\\ 0 & 0 & 0 & \sigma_{v,obs}^{2} \end{array}\right)$$

where symbols with subscripts obs denote variables observed by the roadside sensor, $∆t_{obs}$ denotes the observation interval of the roadside sensor, and N denotes the number of time steps for stabilizing the calculation of the directional angle and velocity. In the simulation, the GT position of the cyclists is used for the observation position.

Next, we will examine the specific parameters of the roadside sensor’s observation. Because the raw LiDAR data is point clouds, the detection process of cyclists is necessary to utilize LiDAR observation in the EKF. Although LiDAR can obtain point clouds precisely, the detection process causes some errors. Thus, we assume $\sigma_{pos,obs}$ as 0.1 m. As for $\sigma_{\theta ,obs}$ and $\sigma_{v,obs}$, we preliminarily determined the values via the numerical simulation. In particular, we added noise, whose average was zero and standard deviation was $\sigma_{pos,obs}$, to the GT position on the assumption that the velocity was 4.2 m/s. Subsequently, we calculated the distribution of θ and v, and determined N, $\sigma_{\theta ,obs}$, and $\sigma_{v,obs}$ as follows:

(33) N=5

(34) $$\sigma_{\theta ,obs}=0.067\;rad$$

(35) $$\sigma_{v,obs}=0.28\;m/s$$

As a result, R is expressed as follows:

(36) $$R=\left(\begin{array}{cccc} \sigma_{pos,obs}^{2} & 0 & 0 & 0\\ 0 & \sigma_{pos,obs}^{2} & 0 & 0\\ 0 & 0 & \sigma_{\theta ,obs}^{2} & 0\\ 0 & 0 & 0 & \sigma_{v,obs}^{2} \end{array}\right)=\left(\begin{array}{cccc} 0.1^{2} & 0 & 0 & 0\\ 0 & 0.1^{2} & 0 & 0\\ 0 & 0 & 0.067^{2} & 0\\ 0 & 0 & 0 & 0.28^{2} \end{array}\right)$$

**Figure 16:**
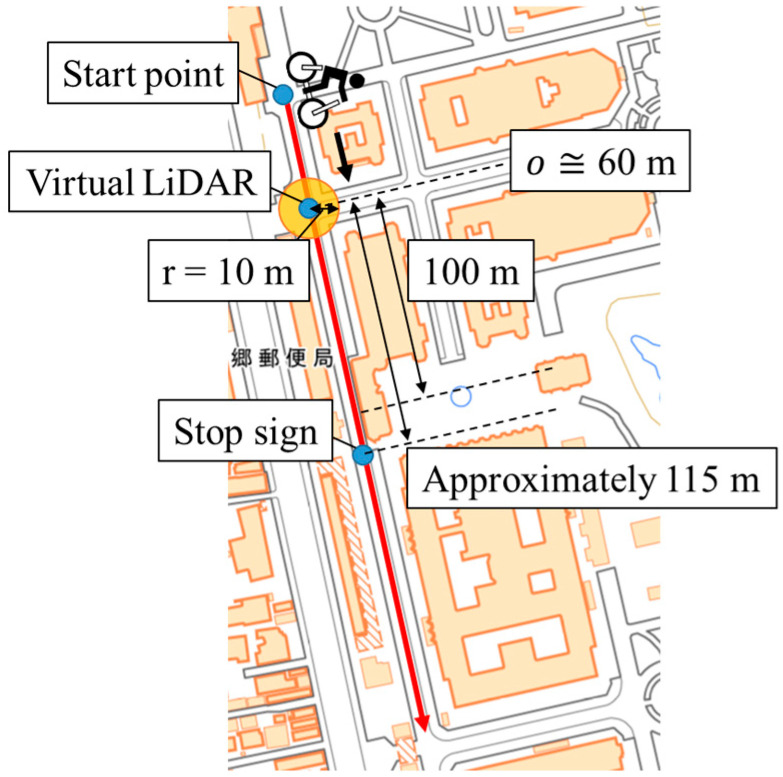
Conceptual schematic of the observation range of the virtual roadside sensor (created by editing the digital map [40]). The Chinese characters on the left side indicate a “post office”.

Finally, we examine the observation range and the observation interval of the roadside sensor. Because the roadside sensor is not actually placed in the LDSI construction data, we set the observation range of the virtual roadside sensor. Figure 16 shows the observation range of the virtual roadside sensor. As shown in Figure 16, we set the virtual roadside sensor to the center of Intersection 1, and set the observation range of the virtual LiDAR as a circular area with a radius of 10 m. As for the observation interval, we set it as 10 Hz from the specification of the actual LiDAR.

#### 4.3.3. Cluster Estimation Results

In the classification evaluation, we first examine the appropriateness of the similarity utilized in the soft classification. To be more precise, we derive the correct classification rate of hard classification, which discretely classifies cyclists using the same score of similarity as the soft classification. Subsequently, we calculate the average classification results of the soft classification method for the data belonging to each cluster and evaluate its performance.

In the classification using the hard classification, 76% of the data were correctly classified. Because hard classification discretizes the similarity score used in the soft classification, we can consider that Equations (8)–(10) used in soft classification appropriately calculate the similarity between the cyclists’ movement characteristics and LDSI of each cluster. Here, LDSI was constructed based on the movement characteristics along the 320 m straight road from the start point to Intersection 2. In contrast, because the observation range of the roadside sensor is a circular area with a radius of 10 m, the information observed only within a range of approximately 20 m is utilized for the classification. In such a condition, the proposed method cannot estimate the cyclists’ cluster correctly when cyclists’ behavior inside the observation range is similar to that of the incorrect cluster. Considering the above condition, the performance of the hard classification is reasonable.

Table 3 shows the average similarity calculated by the soft classification method for the data belonging to each cluster. As shown in Table 3, the soft classification method shows the largest average similarity for the correct cluster. From these results, we can confirm that the soft classification method utilized in this study can estimate cyclists’ clusters with a certain degree of accuracy. However, whether the classification results are useful for the position estimation or not is examined only by the position estimation. The results of the position estimation using the classification results are shown in the following section.

#### 4.3.4. Position Estimation Results

We first explain the estimation results in typical data. From the results, we examine the characteristics of each method. Subsequently, we examine the evaluation metrics of all data and summarize the characteristics of each method.

- Evaluation results in typical data

In the estimation for typical data, we illustrate the estimation results in data A7 and data A14, which belong to Cluster 1. Here, while the classification results of data A7 were relatively good, the classification result of data A14 was inappropriate. The specific results of soft classification for each data are as follows:

- Data A7

$$S_{1}^{\prime }=0.68,S_{2}^{\prime }=0.00,S_{3}^{\prime }=0.32$$

- Data A14

$$S_{1}^{\prime }=0.26,S_{2}^{\prime }=0.35,S_{3}^{\prime }=0.39$$

The HC-based method and SC-based method utilize the above results. Thus, as for the data A14, the HC-based method utilizes the LDSI of the wrong cluster, and the SC-based method utilizes the LDSI of the wrong cluster with relatively high weight.

**Figure 17:**
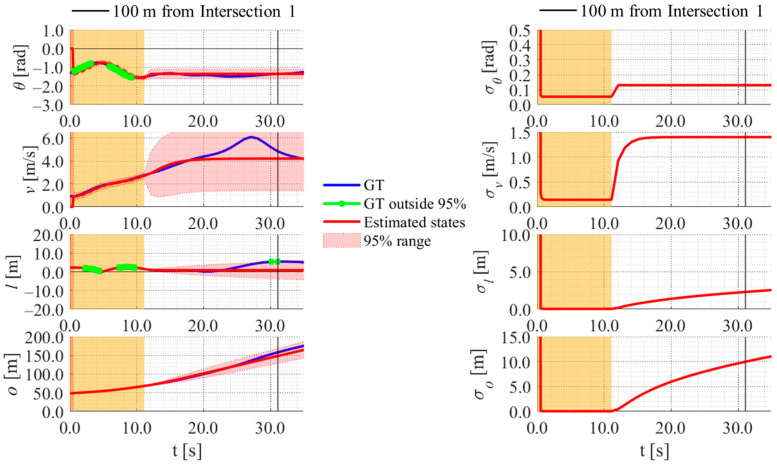
Estimation results of data A7 using the LBSI-based method. The orange areas around 0.0 s indicate the duration during which the cyclists were observed by the roadside sensor.

**Figure 18:**
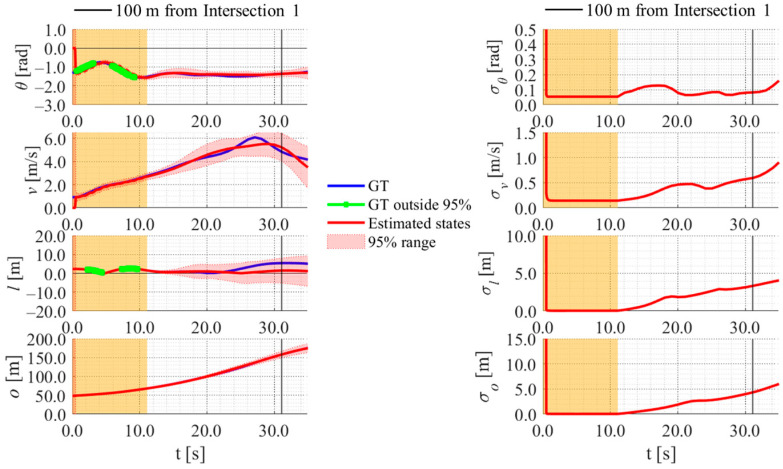
Estimation results of data A7 using the GT cluster-based method. The orange areas around 0.0 s indicate the duration during which the cyclists were observed by the roadside sensor.

**Figure 19:**
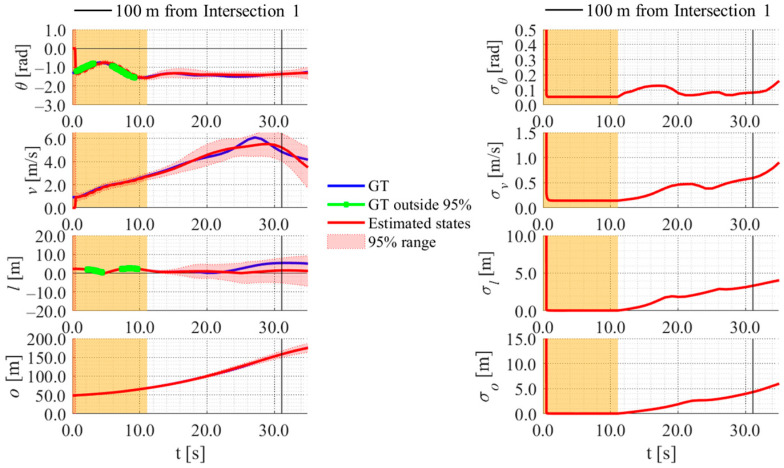
Estimation results of data A7 using the HC-based method. The orange areas around 0.0 s indicate the duration during which the cyclists were observed by the roadside sensor.

**Figure 20:**
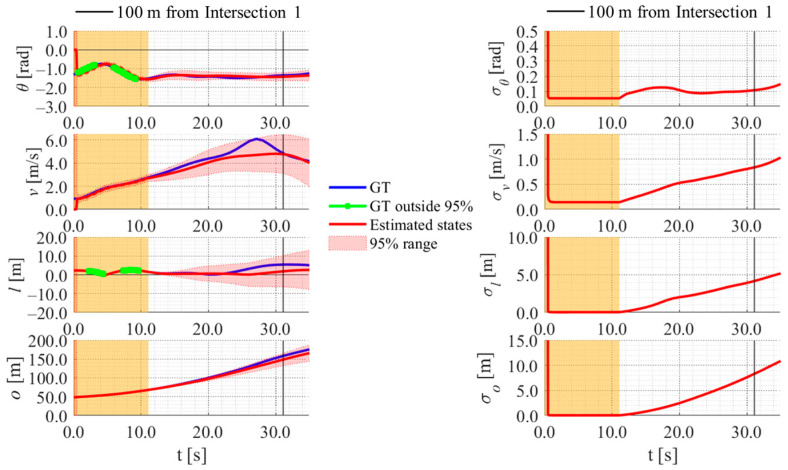
Estimation results of data A7 using the SC-based method. The orange areas around 0.0 s indicate the duration during which the cyclists were observed by the roadside sensor.

**Figure 21:**
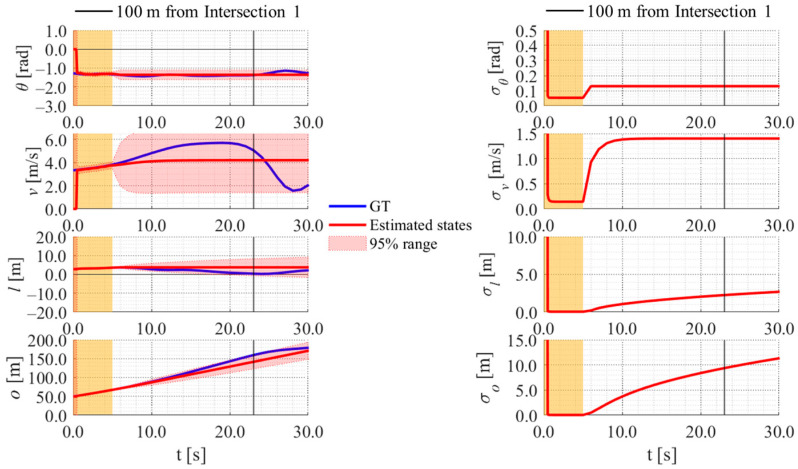
Estimation results of data A14 using the LBSI-based method. The orange areas around 0.0 s indicate the duration during which the cyclists were observed by the roadside sensor.

**Figure 22:**
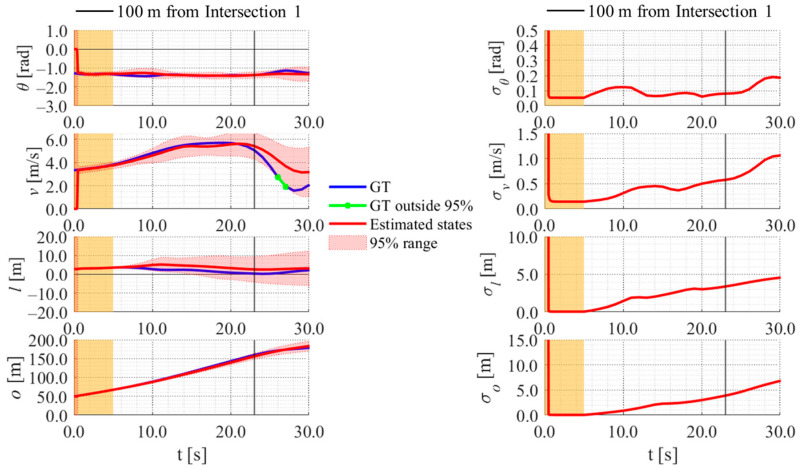
Estimation results of data A14 using the GT cluster-based method. The orange areas around 0.0 s indicate the duration during which the cyclists were observed by the roadside sensor.

**Figure 23:**
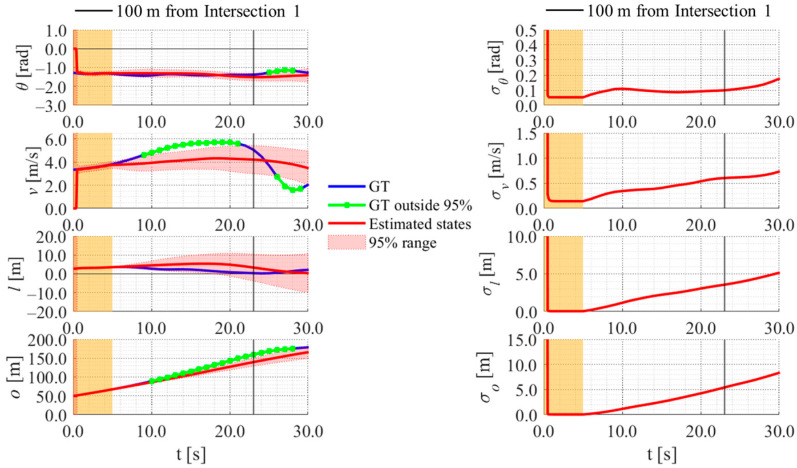
Estimation results of data A14 using the HC-based method. The orange areas around 0.0 s indicate the duration during which the cyclists were observed by the roadside sensor.

**Figure 24:**
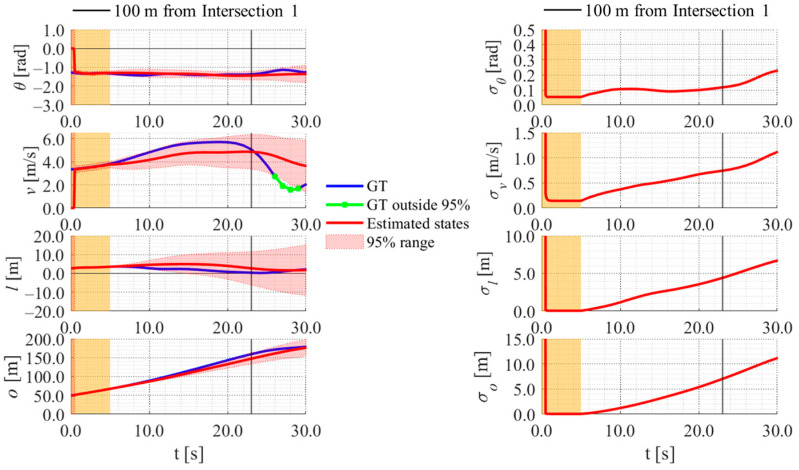
Estimation results of data A14 using the SC-based method. The orange areas around 0.0 s indicate the duration during which the cyclists were observed by the roadside sensor.

Figure 17, Figure 18, Figure 19 and Figure 20 show the estimation results of data A7 by the LBSI-based method, the GT cluster-based method, the HC-based method, and the SC-based method, respectively. Similarly, Figure 21, Figure 22, Figure 23 and Figure 24 show the estimation results of data A14 by each method. In each figure, the left graph shows the estimated variables, the 95%CI, and the GT variables. Blue lines indicate GT variables. The green dots indicate the GT variables that were outside the 95%CI. Red lines indicate the estimated variables, and red areas indicate 95%CI. The orange areas around 0.0 s indicate the duration during which the cyclists were observed by the roadside sensor. The right graph shows the time-series uncertainty related to each variable.

First, we examine the general comparison among the methods. As shown in Figure 17 and Figure 21, when using the LBSI-based method, the directional angle and velocity converged to the average values that were used for VO. In contrast, as shown in Figure 18, Figure 19, Figure 20 and Figure 22, Figure 23 and Figure 24, when using all other methods utilizing LDSI, the estimated velocity changed at each location. In addition, as for the estimated uncertainties shown in Figure 18, Figure 19, Figure 22, and Figure 23, the GT cluster-based methods and the HC-based method showed small values. On the other hand, the LBSI-based methods and the SC-based method showed relatively large values, as shown in Figure 20 and Figure 24.

Next, we examine the estimation results for each data. In data A7, all methods estimated the cyclist’s position appropriately because the GT values are within the 95%CI. In contrast, in data A14, the GT position deviated from the 95%CI in HC-based methods. Because the HC-based method utilized LDSI of the wrong cluster in data A14, the rate of GT offset within the 95%CI was especially smaller than that in other methods. On the other hand, the rates of GT offset within the 95%CI were relatively large for the GT cluster-based method and the SC-based methods. Here, although Cluster 1 showed the smallest similarity in data A14, the SC-based method does not ignore the LDSI of Cluster 1 and utilizes it for position estimation. Therefore, it can be considered that utilizing LDSI based on the soft classification result was effective in data A14.

- Evaluation results of all data

Next, we calculate the evaluation metrics for each method and summarize the estimation results for all data. To be more precise, we conduct position estimation using each method for all data. Then, we calculate the average of the evaluation metrics for each method. Because prevention of crossing collisions is the final goal, the offset, which directly links the performance of predicting collisions, is the most important variable. Thus, we focus only on the offset in the following analyses.

**Figure 25:**
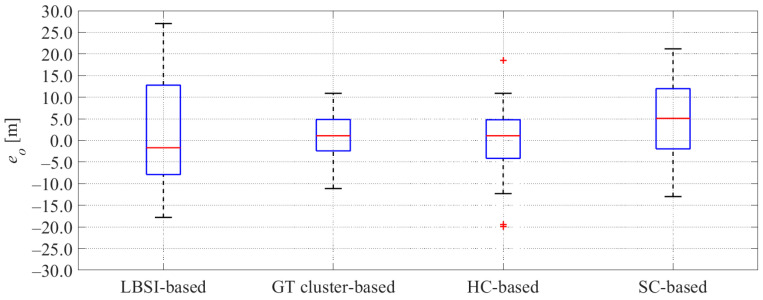
Comparison of estimation error in offset between conventional methods and the proposed method for all data. The red plus signs indicate the outliers. The red lines indicate the median. The blue boxes indicate the interquartile range.

**Figure 26:**
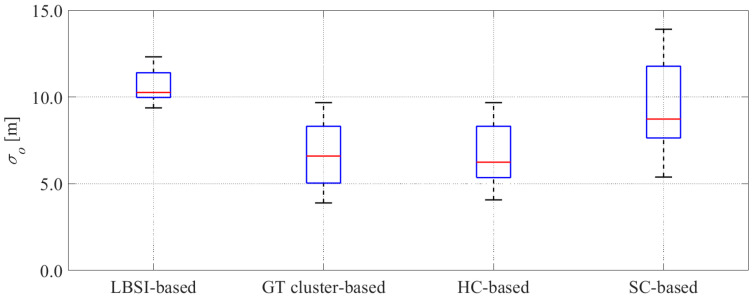
Comparison of estimation uncertainty in offset between conventional methods and the proposed method for all data. The red plus signs indicate the outliers. The red lines indicate the median. The blue boxes indicate the interquartile range.

Figure 25 and Figure 26 show the error and the uncertainties of the estimated offset when the cyclist reached a point 100 m ahead of the roadside sensor in all data. Additionally, Table 4 shows the average rates of the GT offset within the 95%CI, the average absolute error of offset, and the average uncertainty of offset in all data.

As shown in Figure 25 and Figure 26 and Table 4, the GT cluster-based method and the HC-based method can estimate cyclists’ position with small errors and small uncertainties. In contrast, the LBSI-based methods and the SC-based method show relatively large errors and large uncertainties. Here, the errors and uncertainties estimated by the LBSI-based method are larger than those estimated by the SC-based method. Because the smallness of the uncertainty and the rate of the GT values inside 95%CI are in a trade-off relationship, the rate can be improved by employing large uncertainties, even with large errors. Thus, the large uncertainties of the LBSI-based method were the reason for the high rates of the GT offset within the 95%CI.

To evaluate the estimation results, we statistically analyzed the rate within the 95%CI and the absolute error among the LBSI-based method, the HC-based method, and the SC-based method. As for the rate within the 95%CI, first, the Shapiro–Wilk normality test revealed that some of these values include non-normal distribution. Thus, we selected the non-parametric method. Next, Friedman’s test revealed that there is a significant difference in the rate within the 95%CI among the methods. Then, the Wilcoxon signed-rank test with Bonferroni correction revealed that there is a significant difference between the LBSI-based method and the HC-based method. Similarly, we conducted the statistical analysis for the absolute error among the methods. As a result, we found that there are significant differences between the LBSI-based method and the HC-based method, and between the LBSI-based method and the SC-based method.

#### 4.3.5. Discussion

As mentioned in Section 4.2, evaluations from the perspectives of both appropriateness and usefulness are necessary. As for the appropriateness, we evaluate it from the rate of the GT values inside the 95%CI and the estimation errors. As for the usefulness, we evaluate it from the estimation errors and the estimation uncertainties. As shown in Table 4, although the LBSI-based method showed a high average rate inside the 95%CI, its errors and uncertainties were large. Thus, the usefulness of the LBSI-based method is limited. In contrast, the GT cluster-based method showed a relatively high average rate of the GT variables inside the 95%CI, a small average absolute error, and a small average uncertainty. Therefore, the GT cluster-based method can achieve high appropriateness and usefulness. However, because the cyclists’ GT clusters are not identifiable in the real world, the GT cluster-based method is not feasible. As for the HC-based method, the estimation uncertainty is small, while the estimation error is relatively large. As a result, the rate of GT values within the 95%CI is low in the HC-based method. Especially, the rate of GT values within the 95%CI of the HC-based method is significantly lower than that of the LBSI-based method. Therefore, the appropriateness of the HC-based method is low. On the other hand, because the SC-based method considers the classification uncertainty, its estimation uncertainty is moderately large, which can appropriately consider the moderately large error. Therefore, the rate of the GT values within the 95%CI shows a relatively high value. Additionally, the absolute error of the SC-based method is significantly smaller than that of the LBSI-based method. From the above results, we can conclude that the SC-based method achieved high appropriateness, and better usefulness than the LBSI-based method.

### 4.4. Experiment in the Real World

#### 4.4.1. Experimental Setup of the Evaluation in the Real World

In the experimental evaluation of the proposed methods, we conducted an experiment whose setup was similar to the experiment for the data accumulation explained in Section 4.1.1. To be more precise, the experimental participants rode on a bicycle and moved along the experimental course three times. Although the experimental course and the amount of data per participant are the same as those in the experiment in Section 4.1.1, the sensors used for data collection are different. More precisely, the Real Time Kinematic (RTK)-GNSS receiver attached to participants’ helmets was utilized to obtain their accurate position during the experiment. In addition, the RSU equipped with LiDAR was installed in the experimental course to obtain actual observation information. The experiment was conducted in accordance with the Declaration of Helsinki, and the protocol for this experiment was approved by the Research Ethics Committee, School of Engineering, The University of Tokyo (KE25-111) on 8 December 2025. We recruited four experimental participants for this experiment. We obtained written informed consent from the participants. The summary of the data obtained in this experiment is shown in Table 5.

**Figure 27:**
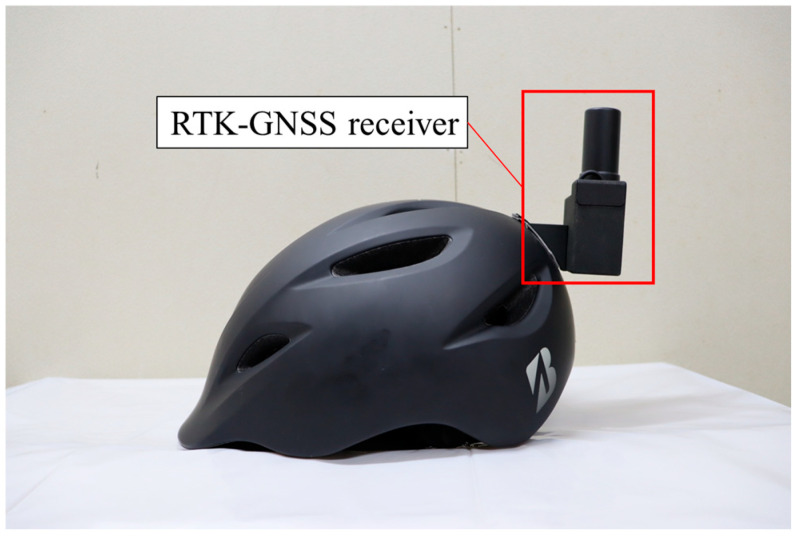
Appearance of the helmet used in the experiment which is equipped with an RTK-GNSS receiver.

**Figure 28:**
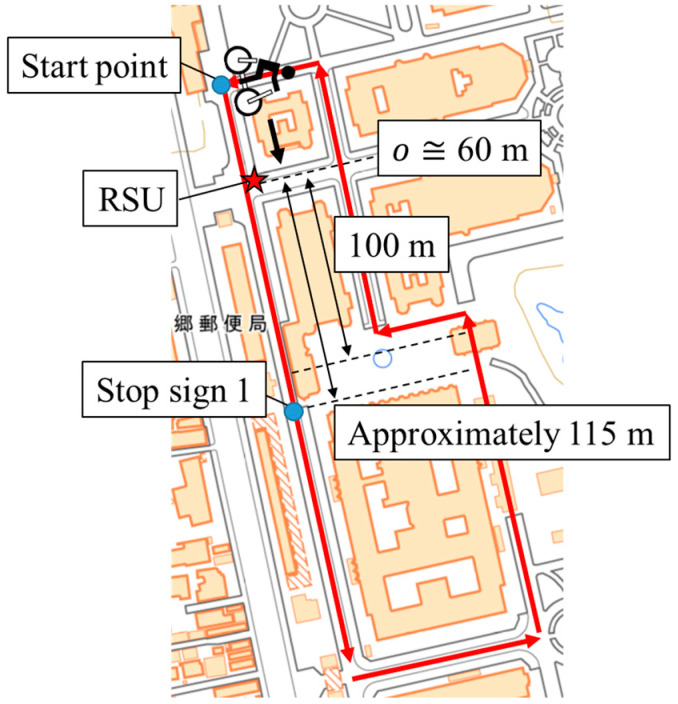
RSU position in the course (created by editing the digital map [40]). The Chinese characters on the left side indicate a “post office”.

**Figure 29:**
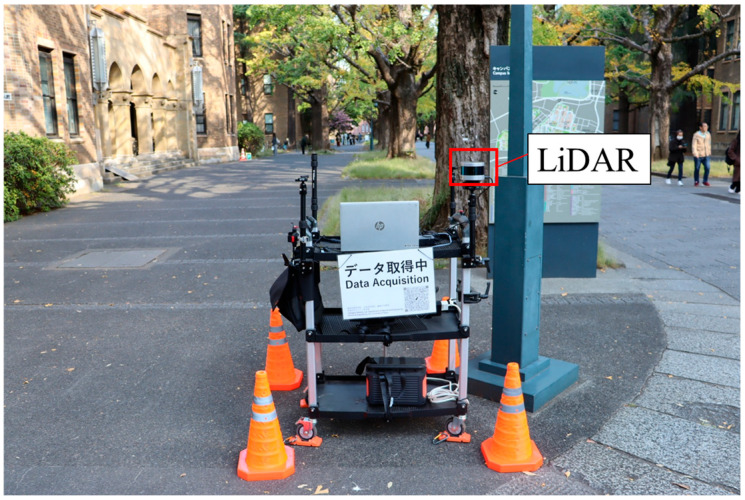
Appearance of the RSU used in the experiment.

In the experiment, the data were recorded utilizing the middleware ROS2 and analyzed afterward. Figure 27 shows the helmet equipped with an RTK-GNSS receiver used in the experiment. The RTK-GNSS data was recorded by a small laptop. Figure 28 shows the RSU position in the course. As shown in Figure 28, the RSU was installed at Intersection 1, which is around position o=60m. Figure 29 shows the appearance of the RSU used in the experiment. The RSU was equipped with the LiDAR VLP-16 [41] and a laptop. The position of the RSU in the global coordinate is obtained by an RTK-GNSS receiver in advance. The directional angle of the RSU in the global coordinate was corrected manually by checking the obtained point cloud of participants and the obtained accurate position of participants. The data obtained by the LiDAR was recorded by the laptop, which was time-synchronized with the small laptop, on the RSU.

In the analysis, we first obtain the participants’ position from the point cloud, which was obtained by the LiDAR. Here, to obtain the cyclist’s position from the point cloud, we removed background points by background filtering [42] and clustered them by DBSCAN [43]. In the background filtering, the grid size was 0.05 m and other parameters were derived automatically based on the method proposed in [42]. In the DBSCAN, the method based on [43] was utilized. To be more precise, we utilized 1.0 m as the searching radius, and all other parameters were derived from predefined subareas and sensor parameters based on [43]. Subsequently, the centroid position of the obtained cluster is transformed into the global coordinate by utilizing the global position of the RSU. The derived position of the participants in the global coordinate is utilized in the EKF. The parameters $\Delta t_{obs}$, N, and R for the roadside sensor’s observation in the EKF are the same as those used in the simulation.

#### 4.4.2. Experimental Results

**Figure 30:**
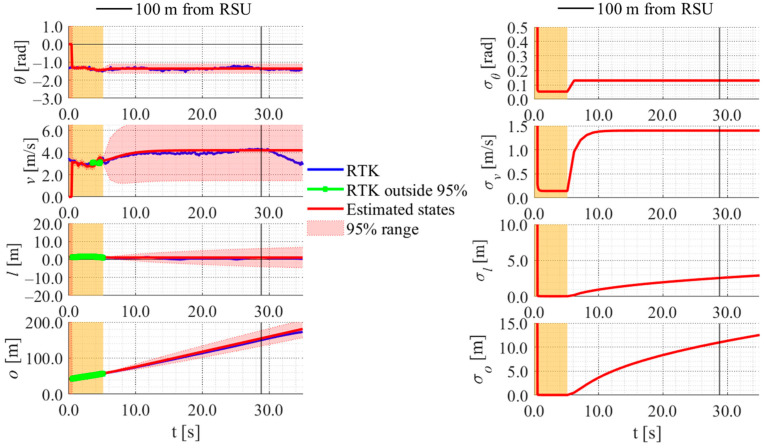
Estimation results of data B4 using the LBSI-based method. The orange areas around 0.0 s indicate the duration during which the cyclists were observed by the roadside sensor.

**Figure 31:**
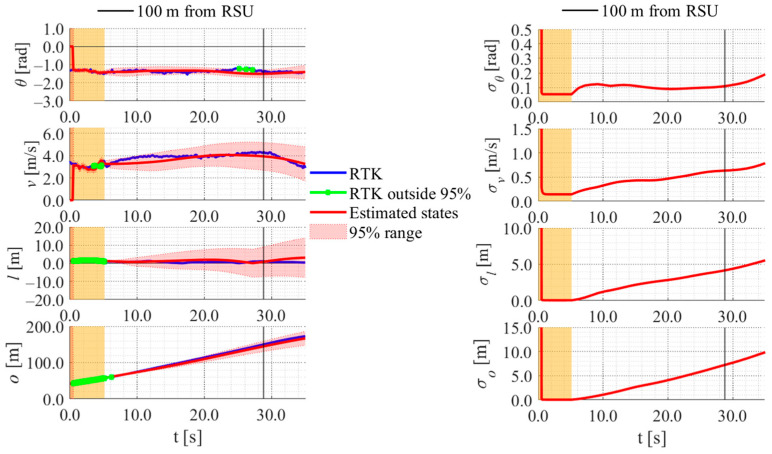
Estimation results of data B4 using the HC-based method. The orange areas around 0.0 s indicate the duration during which the cyclists were observed by the roadside sensor.

**Figure 32:**
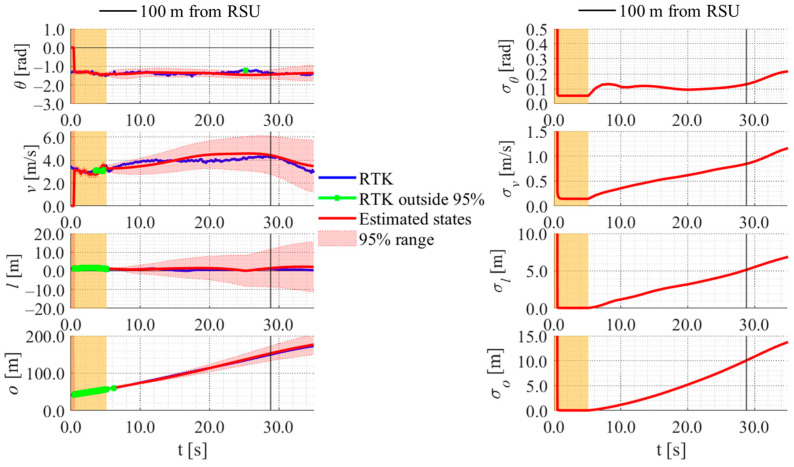
Estimation results of data B4 using the SC-based method. The orange areas around 0.0 s indicate the duration during which the cyclists were observed by the roadside sensor.

Figure 30, Figure 31 and Figure 32 show the estimation results of data B4, which is typical data in the evaluation data, by the LBSI-based method, by the HC-based method, and by the SC-based method, respectively. Similar to the figures in the simulation, the left graphs in each figure show the estimated variables, the 95%CI, and the GT variables. Blue lines indicate variables obtained by the RTK-GNSS receiver. The green dots indicate the variables that were obtained by the RTK-GNSS receiver and were outside the 95%CI. Red lines indicate the estimated variables, and red areas indicate 95%CI. The orange areas around 0.0 s indicate the duration during which the cyclists were observed by the roadside sensor. The right graph in each figure shows the time-series uncertainty related to each variable.

**Figure 33:**
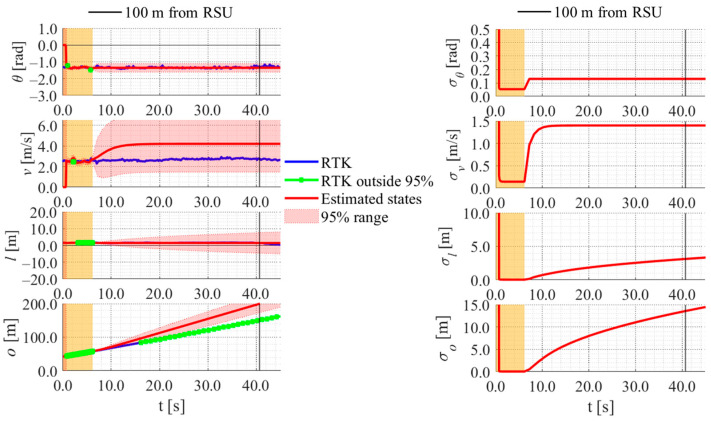
Estimation results of data B1 using the LBSI-based method. The orange areas around 0.0 s indicate the duration during which the cyclists were observed by the roadside sensor.

**Figure 34:**
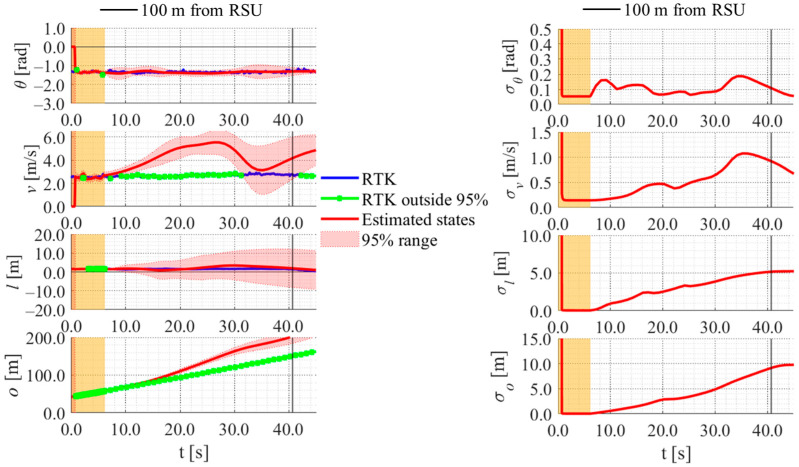
Estimation results of data B1 using the HC-based method. The orange areas around 0.0 s indicate the duration during which the cyclists were observed by the roadside sensor.

As shown in Figure 32, Figure 33 and Figure 34, most of the movement states obtained by the RTK-GNSS receiver is within the 95%CI of estimated states in all methods. Thus, we can consider that all methods can estimate cyclists’ position appropriately in this data. To evaluate the estimation results of each method for all data, we calculate the evaluation metrics used in the simulation evaluation. In the following analysis, similar to the case of the simulation, we focus only on offset, which is most important to prevent crossing collision.

**Figure 35:**
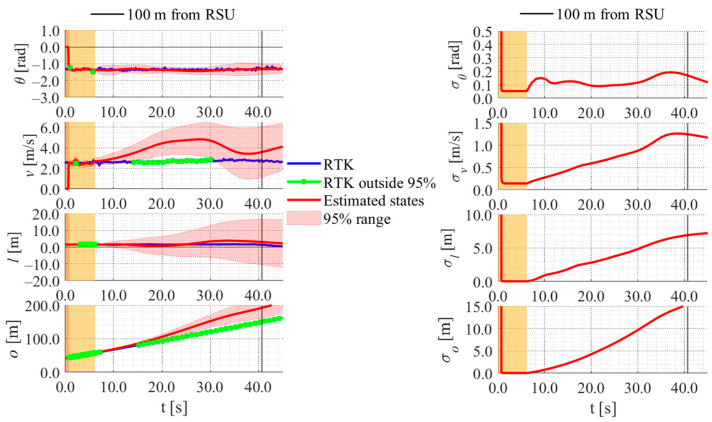
Estimation results of data B1 using the SC-based method. The orange areas around 0.0 s indicate the duration during which the cyclists were observed by the roadside sensor.

Table 6 shows the rates of the offset position obtained by the RTK-GNSS receiver within the 95%CI using each method for all data. Similarly, Table 7 and Table 8 show the error and the uncertainties of the estimated offset when the cyclist reached a point 100 m ahead of the roadside sensor. As shown in Table 6, Table 7 and Table 8, the rates, the errors, and the uncertainties of each method are similar to those in the simulation, except for the data B1–B3. In data B1–B3, the rates are low, and the errors are large across all methods. The reason for such results is the quite low velocity of the experimental participant. To be more precise, the experimental participants in data B1–B3 were the same, and the velocity of the participant was quite low compared to that of the other data used in this study. Figure 33, Figure 34 and Figure 35 show the estimation results of data B1 by each method. As shown in Figure 33, Figure 34 and Figure 35, because the participant moved quite slowly, all methods could not estimate the participant’s offset position appropriately.

#### 4.4.3. Discussions and Limitations

As shown in Table 6, all methods could not estimate the cyclists’ position appropriately in data B1–B3. Here, because the LBSI is not constructed from accumulated data, the LBSI-based method cannot adapt to relatively outlier data. On the other hand, the performance of the HC-based method and the SC-based method can be improved if additional data is accumulated. Therefore, the HC-based method and the SC-based method have the potential to have better robustness against the variety of cyclists’ movement characteristics compared to that of the LBSI-based method.

Because data B1–B3 are unusual data, we evaluate each method based on the estimation results in data B4–B12 in the following discussion. Similar to the results in the simulation, the HC-based method shows small estimated uncertainties. However, because the estimation error was large in certain data, the rate of the values obtained by the RTK-GNSS receiver within the 95%CI was low in the data. Therefore, we can confirm that the robustness of the HC-based method against the classification error was also low in the actual environment.

As for the SC-based method, the average rates of the values obtained by the RTK-GNSS receiver within the 95%CI was 96%. Additionally, the estimated uncertainty of the SC-based method was smaller than that of the LBSI-based method. Thus, in the actual environment, the SC-based method demonstrates high appropriateness while showing better usefulness compared to the LBSI-based method. From the results of the above experimental evaluation and the simulation evaluation, we can confirm that the SC-based method improves the performance of the position estimation by using LDSI and considering classification uncertainty.

However, although the SC-based method showed better performance than conventional methods, there is limitations in the experimental evaluation. Because the data used in the experimental evaluation was obtained from only four experimental participants, the variation in the movement characteristics was limited. Furthermore, for the same reason, the generality of the evaluation result was limited. Similar to the above limitation, because data used for LDSI construction was obtained from only 10 experimental participants, the generality of the constructed LDSI was limited. Therefore, more data for both LDSI construction and the experimental evaluation is desired for a reliable evaluation of the proposed method. Here, the sensitivity of the proposed method to the distribution of the actual movement characteristics, including cases of non-Gaussian distributions, is also desirable. Additionally, because the proposed method was evaluated by using a single RSU’s position, variation in the experimental environment was limited. Thus, an evaluation of the proposed method in various environments is needed. Furthermore, because the experiment in this study was not conducted on an actual community road, evaluation in the actual traffic environment is also necessary.

## 5. Conclusions

In this study, we proposed a position estimation method of cyclists using LDSI, considering classification uncertainty. Subsequently, we evaluated the proposed method in this study through simulation and experiment in the real world. From the evaluations, we confirmed that our proposed method using soft classification estimates cyclists’ position more appropriately than the method using hard classification. We also confirmed that our proposed method shows high appropriateness while showing better usefulness compared to the previously proposed method using LBSI. The proposed method in this study will contribute to the cyclist-related collision prevention around occluded nonsignalized intersections.

However, future research is still needed. Because there are limitations mentioned in Section 4.4.3 in this study, one of the future works is to overcome such limitations. In addition, the proposed method in this study does not discuss the movement changes in the cyclists affected by surrounding environmental changes, such as time of day. Therefore, a position estimation method that can adapt to such a change is necessary. Furthermore, because the experimental participants in this study were conscious of the experiment, their movement characteristics might be different from their daily movement characteristics. Thus, experiments to obtain their actual daily movement characteristics are required. We will try to develop the prototype connected ADASs using the proposed method after the above future works and evaluate the effect of the improvement in the position estimation.

## Figures and Tables

**Table 1:** Comparison of position estimation methods considering mobility diversity. √ indicates positive and x indicates negative.

	ML-Based Methods	Usual Physical-Model-Based Methods	Proposed Methods in Previous Study [18]	Proposed Method in This Study
Consideration of the diversity in the movement characteristics	√	√	√	√
Usability of the observation information in real-time from sensors that is not used in the data accumulation	x	√	√	√
Compensation for the lack of real-time information	x	x	√	√
Consideration of classification uncertainty	√	√	x	√

**Table 2:** Summary of the data for LDSI construction.

Number of participants	10
Number of valid data	29 (Data A1–A29)
Gender	Male: 6, Female: 4
Age	20s~30s

**Table 3:** Average similarity calculated by the soft classification method for the data belonging to each cluster.

GT Cluster	Similarity to Cluster 1	Similarity to Cluster 2	Similarity to Cluster 3
Cluster 1	0.42	0.24	0.34
Cluster 2	0.22	0.62	0.17
Cluster 3	0.31	0.14	0.54

**Table 4:** Average metrics in all data for each method.

Methods		Rate Inside 95% Confidence Interval	Absolute Error [m]	Uncertainty [m]
LBSI-based method		0.97	11.4	10.7
LDSI	GT cluster-based method (Reference)	0.91	4.6	6.6
	HC-based method	0.82	6.4	6.7
	SC-based method	0.91	8.8	9.6

**Table 5:** Summary of the data for experimental evaluation.

Number of participants	4
Number of valid data	12 (Data B1–B12)
Gender	Male: 1, Female: 3
Age	20s~30s

**Table 6:** Rates of the offset observed by RTK-GNSS within the 95%CI.

Data ID	LBSI-Based Method	HC-Based Method	SC-Based Method
B1	0.26	0.00	0.21
B2	0.29	0.00	0.26
B3	0.21	0.42	0.63
B4	1.00	0.96	0.96
B5	1.00	1.00	1.00
B6	1.00	1.00	1.00
B7	1.00	0.96	0.96
B8	1.00	0.04	0.92
B9	1.00	0.95	0.95
B10	1.00	0.96	0.96
B11	1.00	0.95	0.95
B12	1.00	0.95	0.95
Average (data B1–B12)	0.81	0.68	0.81
Average (data B4–B12)	1.00	0.86	0.96

**Table 7:** Error of the offset estimation in meters.

Data ID	LBSI-Based Method	HC-Based Method	SC-Based Method
B1	48.9	51.4	41.9
B2	49.1	50.5	39.4
B3	57.7	53.2	42.1
B4	4.8	−5.5	3.0
B5	0.0	7.4	8.3
B6	5.6	−0.9	7.5
B7	8.0	−4.5	4.4
B8	7.5	15.5	10.8
B9	2.2	9.9	5.8
B10	22.5	3.6	12.7
B11	1.1	−7.5	−0.1
B12	−1.2	−7.9	0.2
Average of absolute value (data B1–B12)	17.4	18.1	14.7
Average of absolute value (data B4–B12)	5.9	7.0	5.9

**Table 8:** Uncertainty of the offset estimation in meters.

Data ID	LBSI-Based Method	HC-Based Method	SC-Based Method
B1	13.6	9.1	15.4
B2	13.6	9.1	15.6
B3	14.6	12.3	19.3
B4	11.0	7.2	10.1
B5	10.5	5.1	7.8
B6	11.0	7.3	10.2
B7	11.2	7.5	10.6
B8	11.1	6.0	10.5
B9	10.8	5.4	9.7
B10	12.2	8.9	12.8
B11	10.8	7.0	9.6
B12	10.6	6.7	9.3
Average (data B1–B12)	11.7	7.6	11.7
Average (data B4–B12)	11.0	6.8	10.1

**Figure A1:**
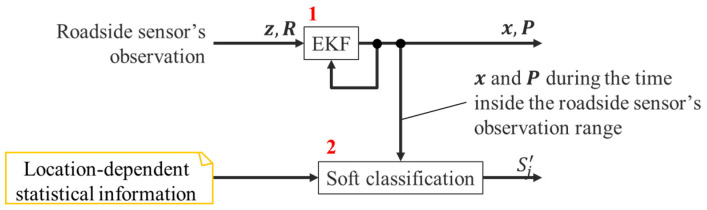
Overview of the calculation process 1 and 2 in proposed method.

**Figure A2:**
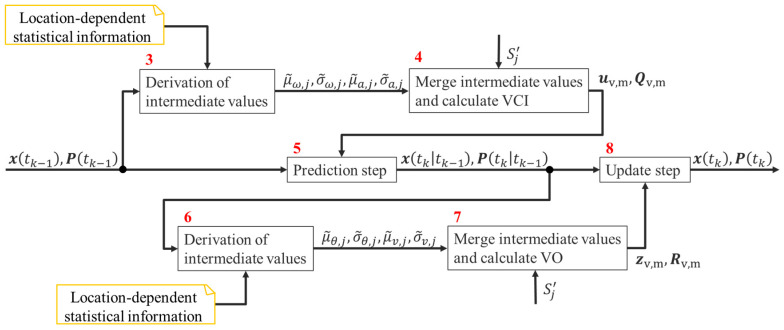
Overview of the calculation process 3–8 in proposed method.

## Data Availability

The original contributions presented in this study are included in the article.

## Abbreviations

The following abbreviations are used in this manuscript:

LDSI	Location-Dependent Statistical Information
VO	Virtual Observation
VCI	Virtual Control Input
ADAS	Advanced Driver Assistance System
LBSI	Literature-Based Statistical Information
GNSS	Global Navigation Satellite System
RSU	RoadSide Unit
LiDAR	Light Detection And Ranging
ML	Machine Learning
IMM	Interacting Multiple Model
EKF	Extended Kalman filter
LD	Lateral Deviation
CI	Confidence Interval
SC	Soft Classification
GT	Ground Truth
HC	Hard Classification
RTK	Real Time Kinematic

## Appendix A. VO and VCI

Here, we explain details of the VO and the VCI based on our previous study [18]. In the calculation of the VO and the VCI, we first derive intermediate values $\tilde{\mu }$ and $\tilde{\sigma }$ by using LDSI, the cyclists’ cluster, and the estimated cyclists’ position. Here, because the cyclists’ position is estimated value, its uncertainty is also considered in the calculation of the intermediate values. The VCI and its uncertainty are derived from the intermediate values corresponding to the acceleration and the yawing rate. Similarly, the VO is derived from the intermediate values corresponding to the velocity and the directional angle. As for the uncertainty of the VO, the time-series effect should be considered for appropriate estimation. In general, the estimated uncertainty of the EKF converges to a smaller value than the corresponding value of observation uncertainty. Thus, we derive and utilize the uncertainty of the VO which converges the estimated uncertainty to the intermediate values corrected by safety factor.

The VCI $u_{v}$ and the VO $z_{v}$ are expressed as Equations (11)–(13). As shown in Equation (11), the intermediate values calculated from estimated cyclists’ position at $t_{k-1}$, which is the latest estimated position, is used for the VCI $u_{v}$. On the other hand, as shown in Equation (12), the intermediate values calculated from estimated cyclists’ position at $t_{k}$ is used for the VO $z_{v}$. Here, the cyclists’ position at $t_{k}$ is predicted using latest estimated positon and VCI. In the calculation of the uncertainty of VCI $Q_{v}$ and the uncertainty of VO $R_{v}$, we correct intermediate values by the safety factors ${sf}_{Q}$ and ${sf}_{R}$. The intermediate values which are corrected by safety factors are expressed as follows:

(A1) $${\tilde{\sigma }}_{\theta }^{\prime }\left(t_{k}\right)=\left(1+{sf}_{R}\right){\tilde{\sigma }}_{\theta }\left(t_{k}\right)$$

(A2) $${\tilde{\sigma }}_{v}^{\prime }\left(t_{k}\right)=\left(1+{sf}_{R}\right){\tilde{\sigma }}_{v}\left(t_{k}\right)$$

(A3) $${\tilde{\sigma }}_{\omega }^{\prime }\left(t_{k-1}\right)=\left(1+{sf}_{Q}\right){\tilde{\sigma }}_{\omega }\left(t_{k-1}\right)$$

(A4) $${\tilde{\sigma }}_{a}^{\prime }\left(t_{k-1}\right)=\left(1+{sf}_{Q}\right){\tilde{\sigma }}_{a}\left(t_{k-1}\right)$$

where $\tilde{\sigma }$ with subscripts denote the intermediate values before the correction and ${\tilde{\sigma }}^{\prime }$ with subscripts denote the intermediate values after the correction. Using the corrected intermediate values above, $Q_{v}$ is expressed as Equation (14) and $R_{v}$ is expressed as follows:

(A5) $$\begin{array}{c} R_{v}\left(t_{k}\right)=g\left({{\tilde{\sigma }}_{\theta }^{\prime }}^{2}\left(t_{k}\right),{{\tilde{\sigma }}_{v}^{\prime }}^{2}\left(t_{k}\right),{\tilde{\sigma }}_{\omega }^{\prime }\left(t_{k-1}\right),{\tilde{\sigma }}_{a}^{\prime }\left(t_{k-1}\right),∆t\right)\\ =\left(\begin{array}{cc} {\left[\frac{\sqrt{{{\tilde{\sigma }}_{\theta }^{\prime }}^{4}\left(t_{k}\right)+{{\tilde{\sigma }}_{\omega }^{\prime }}^{2}\left(t_{k-1}\right)∆t^{2}{{\tilde{\sigma }}_{\theta }^{\prime }}^{2}\left(t_{k}\right)}}{{\tilde{\sigma }}_{\omega }^{\prime }\left(t_{k-1}\right)∆t}\right]}^{2} & 0\\ 0 & {\left[\frac{\sqrt{{{\tilde{\sigma }}_{v}^{\prime }}^{4}\left(t_{k}\right)+{{\tilde{\sigma }}_{a}^{\prime }}^{2}\left(t_{k-1}\right)∆t^{2}{{\tilde{\sigma }}_{v}^{\prime }}^{2}\left(t_{k}\right)}}{{\tilde{\sigma }}_{a}^{\prime }\left(t_{k-1}\right)∆t}\right]}^{2} \end{array}\right) \end{array}$$

By using the uncertainty calculated by above equations, the uncertainty of the corresponding states converges to the ${\tilde{\sigma }}_{\theta }^{\prime }$ and ${\tilde{\sigma }}_{v}^{\prime }$.

## Appendix B. Overall Calculation Process

Here, we explain details of the overall calculation process. The overall calculation process is organized as follows.

1. Position estimation using RSU observation.
2. Conduct soft classification using Equations (8)–(10) when cyclists exit the observation range of RSU.

(The following processes are repeated at a certain interval.)

3. Derivation of intermediate values corresponding to acceleration and yawing rate for each cluster from latest estimated position.
4. Merge intermediate values and calculate VCI using Equations (20), (22)–(24).
5. Conduct prediction step of EKF using VCI.
6. Calculate intermediate values corresponding to velocity and directional angle for each cluster from predicted position.
7. Merge intermediate values and calculate VO using Equations (21), (25)–(27).
8. Conduct update step of EKF using VO.

The flow chart of above process from 1 and 2 is shown in Figure A1. The flow chart of above process from 3 to 8 is shown in Figure A2. In the Figure A2, Location dependent statistical information used in the process 3 is same as that in the process 6. Similarly, $S_{j}^{\prime }$ used in the process 4 is same as those in the process 7.

## References

[1] Cabinet Office White Paper on Traffic Safety in Japan 2025. https://www8.cao.go.jp/koutu/taisaku/r07kou_haku/english/pdf/wp2025.pdf.

[2] Cabinet Office White Paper on Traffic Safety in Japan 2021. https://www8.cao.go.jp/koutu/taisaku/r03kou_haku/english/pdf/wp2021-1.pdf.

[3] National Police Agency Annual Report by Traffic Accidents 2025 Trends in Traffic Accidents by Type of Accident. https://www.npa.go.jp/publications/statistics/koutsuu/toukeihyo_e.html.

[4] Bo Bo N., Slembrouck M., Veelaert P., Philips W. (2020). Distributed Multi-Class Road User Tracking in Multi Camera Network for Smart Traffic Applications. Advanced Concepts for Intelligent Vision Systems.

[5] Zhang J., Xiao W., Coifman B., Mills J.P. (2020). Vehicle Tracking and Speed Estimation from Roadside Lidar. IEEE J. Sel. Top. Appl. Earth Obs. Remote Sens..

[6] Zhang R., Zou Z., Shen S., Liu H.X. (2022). Design, Implementation, and Evaluation of a Roadside Cooperative Perception System. Transp. Res. Rec..

[7] Sugimoto C., Nakamura Y., Hashimoto T. Prototype of pedestrian-to-vehicle communication system for the prevention of pedestrian accidents using both 3G wireless and WLAN communication. Proceedings of the 2008 3rd International Symposium on Wireless Pervasive Computing.

[8] Liu W., Muramatsu S., Okubo Y. Cooperation of V2I/P2I Communication and Roadside Radar Perception for the Safety of Vulnerable Road Users. Proceedings of the 2018 16th International Conference on Intelligent Transportation Systems Telecommunications (ITST).

[9] Hussein A., García F., Armingol J.M., Olaverri-Monreal C. P2V and V2P communication for Pedestrian warning on the basis of Autonomous Vehicles. Proceedings of the 2016 IEEE 19th International Conference on Intelligent Transportation Systems (ITSC).

[10] Ito T. (2025). Connected Collision Avoidance System via Stochastic Localization on Community Roads. Int. J. Automot. Eng..

[11] Alouani A.T., Blair W.D. (1993). Use of a kinematic constraint in tracking constant speed, maneuvering targets. IEEE Trans. Autom. Control.

[12] Zhou G., Li K., Kirubarajan T., Xu L. (2019). State Estimation with Trajectory Shape Constraints Using Pseudomeasurements. IEEE Trans. Aerosp. Electron. Syst..

[13] Zhang Z., Li K., Zhou G. (2022). State Estimation with Heading Constraints for On-Road Vehicle Tracking. IEEE Trans. Intell. Transp. Syst..

[14] Vargas-Meléndez L., Boada B.L., Boada M.J.L., Gauchía A., Díaz V. (2016). A Sensor Fusion Method Based on an Integrated Neural Network and Kalman Filter for Vehicle Roll Angle Estimation. Sensors.

[15] Kim D., Kim G., Choi S., Huh K. (2021). An Integrated Deep Ensemble-Unscented Kalman Filter for Sideslip Angle Estimation with Sensor Filtering Network. IEEE Access.

[16] Jiang Y., Nong X. A Radar Filtering Model for Aerial Surveillance Base on Kalman Filter and Neural Network. Proceedings of the 2020 IEEE 11th International Conference on Software Engineering and Service Science (ICSESS).

[17] Suzuki K., Watanabe K., Yang J., Ito T. (2025). Virtual Observation Using Statistical Information of Cyclist’s Velocity for Estimation of Position and Uncertainty. Int. J. Automot. Eng..

[18] Suzuki K., Ito T. (2025). Virtual Observation Using Location-Dependent Statistical Information of Cyclists’ Movement for Estimation of Position and Uncertainty. Sensors.

[19] Eriksson J., Forsman Å., Niska A., Gustafsson S., Sörensen G. (2019). An analysis of cyclists’ speed at combined pedestrian and cycle paths. Traffic Inj. Prev..

[20] Clarry A., Imani A.F., Miller E.J. (2019). Where we ride faster? Examining cycling speed using smartphone GPS data. Sustain. Cities Soc..

[21] Twisk D., Stelling A., Van Gent P., De Groot J., Vlakveld W. (2021). Speed characteristics of speed pedelecs, pedelecs and conventional bicycles in naturalistic urban and rural traffic conditions. Accid. Anal. Prev..

[22] Yan H., Maat K., van Wee B. (2025). Cycling speed variation: A multilevel model of characteristics of cyclists, trips and route tracking points. Transportation.

[23] Bernhoft I.M., Carstensen G. (2008). Preferences and behaviour of pedestrians and cyclists by age and gender. Transp. Res. Part F Traffic Psychol. Behav..

[24] O’Hern S., Stephens A.N., Young K.L., Koppel S. (2020). Personality traits as predictors of cyclist behaviour. Accid. Anal. Prev..

[25] Oehl M., Brandenburg S., Huemer A.K. (2019). Cyclists’ anger experiences in traffic: The Cycling Anger Scale. Transp. Res. Part F Traffic Psychol. Behav..

[26] Li D., Chen X., Huang K. Multi-attribute learning for pedestrian attribute recognition in surveillance scenarios. Proceedings of the 2015 3rd IAPR Asian Conference on Pattern Recognition (ACPR).

[27] Li D., Chen X., Zhang Z., Huang K. Pose Guided Deep Model for Pedestrian Attribute Recognition in Surveillance Scenarios. Proceedings of the 2018 IEEE International Conference on Multimedia and Expo (ICME).

[28] Liu X., Zhao H., Tian M., Sheng L., Shao J., Yi S., Yan J., Wang X. HydraPlus-Net: Attentive Deep Features for Pedestrian Analysis. Proceedings of the 2017 IEEE International Conference on Computer Vision (ICCV).

[29] Zeng H., Ai H., Zhuang Z., Chen L. Multi-Task Learning via Co-Attentive Sharing for Pedestrian Attribute Recognition. Proceedings of the 2020 IEEE International Conference on Multimedia and Expo (ICME).

[30] Horn M., Schumann O., Hahn M., Dickmann J., Dietmayer K. Motion Classification and Height Estimation of Pedestrians Using Sparse Radar Data. Proceedings of the 2018 Sensor Data Fusion: Trends, Solutions, Applications (SDF).

[31] Shen C., Chao F., Wu W., Wang R., Huang G.Q., Yu S. LidarGait: Benchmarking 3D Gait Recognition with Point Clouds. Proceedings of the 2023 IEEE/CVF Conference on Computer Vision and Pattern Recognition (CVPR).

[32] Chai Y., Sapp B., Bansal M., Anguelov D. (2019). MultiPath: Multiple Probabilistic Anchor Trajectory Hypotheses for Behavior Prediction. arXiv.

[33] Abdelraouf A., Gupta R., Han K. Interaction-Aware Personalized Vehicle Trajectory Prediction Using Temporal Graph Neural Networks. Proceedings of the 2023 IEEE 26th International Conference on Intelligent Transportation Systems (ITSC).

[34] Li X., Zhang M., Tavares A.J., Xu H., Ma S. (2025). Personalized vehicle trajectory prediction method based on driving style classification. Sci. Rep..

[35] Chen G., Li J., Zhou N., Ren L., Lu J. Personalized Trajectory Prediction via Distribution Discrimination. Proceedings of the 2021 IEEE/CVF International Conference on Computer Vision (ICCV).

[36] Dyckmanns H., Matthaei R., Maurer M., Lichte B., Effertz J., Stüker D. Object tracking in urban intersections based on active use of a priori knowledge: Active interacting multi model filter. Proceedings of the 2011 IEEE Intelligent Vehicles Symposium (IV).

[37] Jeong Y., Yi K. (2021). Target Vehicle Motion Prediction-Based Motion Planning Framework for Autonomous Driving in Uncontrolled Intersections. IEEE Trans. Intell. Transp. Syst..

[38] Yi D., Su J., Liu C., Chen W.-H. (2019). Trajectory Clustering Aided Personalized Driver Intention Prediction for Intelligent Vehicles. IEEE Trans. Ind. Inform..

[39] Ito T., Mio M., Tohriyama K., Kamata M. (2016). Novel Map Platform Based on Primitive Elements of Traffic Environments for Automated Driving Technologies. Int. J. Automot. Eng..

[40] Geospatial Information Authority of Japan GSI Maps. https://maps.gsi.go.jp/.

[41] OUSTER Puck Datasheet. https://ouster.com/products/hardware/vlp-16.

[42] Wu J., Xu H., Sun Y., Zheng J., Yue R. (2018). Automatic Background Filtering Method for Roadside LiDAR Data. Transp. Res. Rec..

[43] Zhao J., Xu H., Liu H., Wu J., Zheng Y., Wu D. (2019). Detection and tracking of pedestrians and vehicles using roadside LiDAR sensors. Transp. Res. Part C Emerg. Technol..

